# Supplementary material for: Machine learning optimized polygenic scores for blood cell traits identify sex-specific trajectories and genetic correlations with disease
Source: Cell Genom. 2022 Jan 13;2(1):100086. doi: 10.1016/j.xgen.2021.100086 (PMC8758502; doi:10.1016/j.xgen.2021.100086)

Cell Genomics, Volume *2*

Supplemental information

Machine learning optimized polygenic scores for blood cell traits identify sex-specific trajectories and genetic correlations with disease

Yu Xu, Dragana Vuckovic, Scott C. Ritchie, Parsa Akbari, Tao Jiang, Jason Grealey, Adam S. Butterworth, Willem H. Ouwehand, David J. Roberts, Emanuele Di Angelantonio, John Danesh, Nicole Soranzo, and Michael Inouye

Supplementary Tables and Figures

Table S1. Summary of measurement methods for the 26 blood cell traits, related to STAR Methods

Cell Type	Standard Abbreviation	Long Name	Unit	Description	Coulter LH 700 Series (UK Biobank)		Sysmex XN-1000 (INTERVAL)	
					Measured / Derived	Determination	Measured / Derived	Determination
Platelet	PLT#	Platelet count	per nL	Count of platelets per unit volume of blood	Measured	Impedance	Measured	Flow cytometry gate (impedance for missing data points)
	MPV	Mean platelet volume	fL	Mean volume of platelets	Derived	(PCT/PLT#)×10000	Derived	(PCT/PLT#)×10000
	PDW	Platelet distribution width	fL	The spread of the platelet volume distribution. Note that Sysmex and Coulter use different statistics to measure spread.	Measured	Impedance: Coefficient of variation of platelet volume distribution	Measured	Impedance: width at 20% peak height of platelet volume histogram.
	PCT	Plateletcrit	%	Volume fraction of blood occupied by platelets	Measured	Impedance	Measured	Impedance
Mature red cell	RBC#	Red blood cell count	per pL	Count of red blood cells per unit volume of blood	Measured	Impedance	Measured	Impedance
	MCV	Mean corpuscular volume	fL	Mean volume of red blood cells	Derived	(HCT/RBC#)×10	Derived	(HCT/RBC#)×10
	HCT	Hematocrit	%	Volume fraction of blood occupied by red cells	Measured	Impedance	Measured	Impedance
	MCH	Mean corpuscular hemoglobin	pg	Average mass of hemoglobin per red cell	Derived	(HGB/RBC#)×10	Derived	(HGB/RBC#)×10
	MCHC	Mean corpuscular hemoglobin concentration	g/dL	Concentration of hemoglobin with respect to unit of volume occupied by red cells	Derived	(HGB/HCT)×100	Derived	(HGB/HCT)×100
	HGB	Hemoglobin concentration	g/dL	Concentration of hemoglobin with respect to unit of volume of blood	Measured	Light absorbance	Measured	Light absorbance
Immature red cell	RET#	Reticulocyte count	pL	Count of reticulocytes per unit volume of blood	Derived	(RET%×RBC#)/100	Derived	(RET%×RBC#)/100
	RET%	Reticulocyte fraction of red cells	%	Percentage of red blood cells that are reticulocytes	Measured	Flow cytometry/impedance	Measured	Flow cytometry gates
	IRF	Immature fraction of reticulocytes	-	Fraction of reticulocytes with high RNA content, as measured by light scatter	Derived	HLSR#/RET#	Measured	Flow cytometry gates

Cell Type	Standard Abbreviation	Long Name	Unit	Description	Coulter LH 700 Series (UK Biobank)		Sysmex XN-1000 (INTERVAL)	
					Measured / Derived	Determination	Measured / Derived	Determination
Immature red cell	HLSR#	High light scatter reticulocyte count	per pL	Count of high RNA content (immature) reticulocytes per unit volume of blood	Derived	(HLSR%×RBC#)/100%	Derived	IRF×RET#
	HLSR%	High light scatter reticulocyte percentage of red cells	%	Immature reticulocyte count as a percentage of red blood cell count	Measured	Flow cytometry/impedance gates	Derived	(HLSR#/RBC#)×100%
Myeloid white cell	MONO#	Monocyte count	per nL	Count of monocytes per unit volume of blood	Derived	(MONO%×WBC#)/100%	Derived	(MONO%×WBC#)/100%
	NEUT#	Neutrophil count	per nL	Count of neutrophils per unit volume of blood	Derived	(NEUT%×WBC#)/100%	Derived	(NEUT%×WBC#)/100%
	EO#	Eosinophil count	per nL	Count of eosinophils per unit volume of blood	Derived	(EO%×WBC#)/100%	Derived	(EO%×WBC#)/100%
	BASO#	Basophil count	per nL	Count of basophils per unit volume of blood	Derived	(BASO%×WBC#)/100%	Derived	(BASO%×WBC#)/100%
Lymphoid white cell	LYMPH#	Lymphocyte count	per nL	Aggregate count of lymphoid cells per unit volume of blood	Derived	(LYMPH%×WBC#)/100%	Derived	(LYMPH%×WBC#)/100%
Compound white cell	WBC#	White blood cell count	per nL	Aggregate count of white cells per unit volume of blood	Measured	Impedance	Measured	Flow cytometry gates
	MONO%	Monocyte percentage of white cells	%	Percentage of white cells that are monocytes	Measured	Flow cytometry gates	Measured	Flow cytometry gates
	NEUT%	Neutrophil percentage of white cells	%	Percentage of white cells that are neutrophils	Measured	Flow cytometry gates	Measured	Flow cytometry gates
	EO%	Eosinophil percentage of white cells	%	Percentage of white cells that are eosinophils	Measured	Flow cytometry gates	Measured	Flow cytometry gates
	BASO%	Basophil percentage of white cells	%	Percentage of white cells that are basophils	Measured	Flow cytometry gates	Measured	Flow cytometry gates
	LYMPH%	Lymphocyte percentage of white cells	%	Percentage of white cells that are lymphocytes	Measured	Flow cytometry gates	Measured	Flow cytometry gates

Table S2. The number of samples and conditional analysis variants used in UK biobank and INTERVAL for each blood cell trait, related to STAR Methods. This table presents the number of valid samples after quality control and the number of variants selected in conditional analysis for each trait.

Trait	Number of Valid Samples		Number of Variants
	UK Biobank	INTERVAL	
PLT#	391232	38939	762
MPV	391598	37224	681
PDW	391450	37262	579
PCT	390803	37306	726
RBC#	408069	40262	707
MCV	407157	40080	739
HCT	408112	40340	513
MCH	406517	40108	682
MCHC	407850	40265	252
HGB	407739	40329	532
RET#	396720	40253	590
RET%	396811	40286	572
IRF	396408	40227	390
HLSR#	400334	40244	605
HLSR%	400438	40225	594
MONO#	403994	39177	674
NEUT#	406788	39138	512
EO#	406470	40276	623
BASO#	404718	39986	198
LYMPH#	407277	39191	639
WBC#	408032	40466	659
MONO%	403136	39189	583
NEUT%	407114	39190	452
EO%	406417	40326	589
BASO%	404532	40133	160
LYMPH%	407319	39178	489

Figure S1. Comparison of CA variant effect sizes between GWAS and EN/BR method, related to Figure 2. EN and BR generated almost the same effect sizes for conditional analysis variants of all the traits, thus for simplicity, this figure only compares the variant effect sizes between EN and the univariant analysis in GWAS. The mean of the 5 effect sizes in the 5 trained EN models for each variant is used as the variant effect size of EN in this figure. The variants whose MAF is smaller than 1% are marked with triangles and others are marked with circles. Those variants that were detected with interactions are marked in red, and variants that were correlated with others with $r^2 > 0.1$ are marked in yellow. If variants fall in both of the scenarios, they are marked in green. Any other variants are marked in blue.

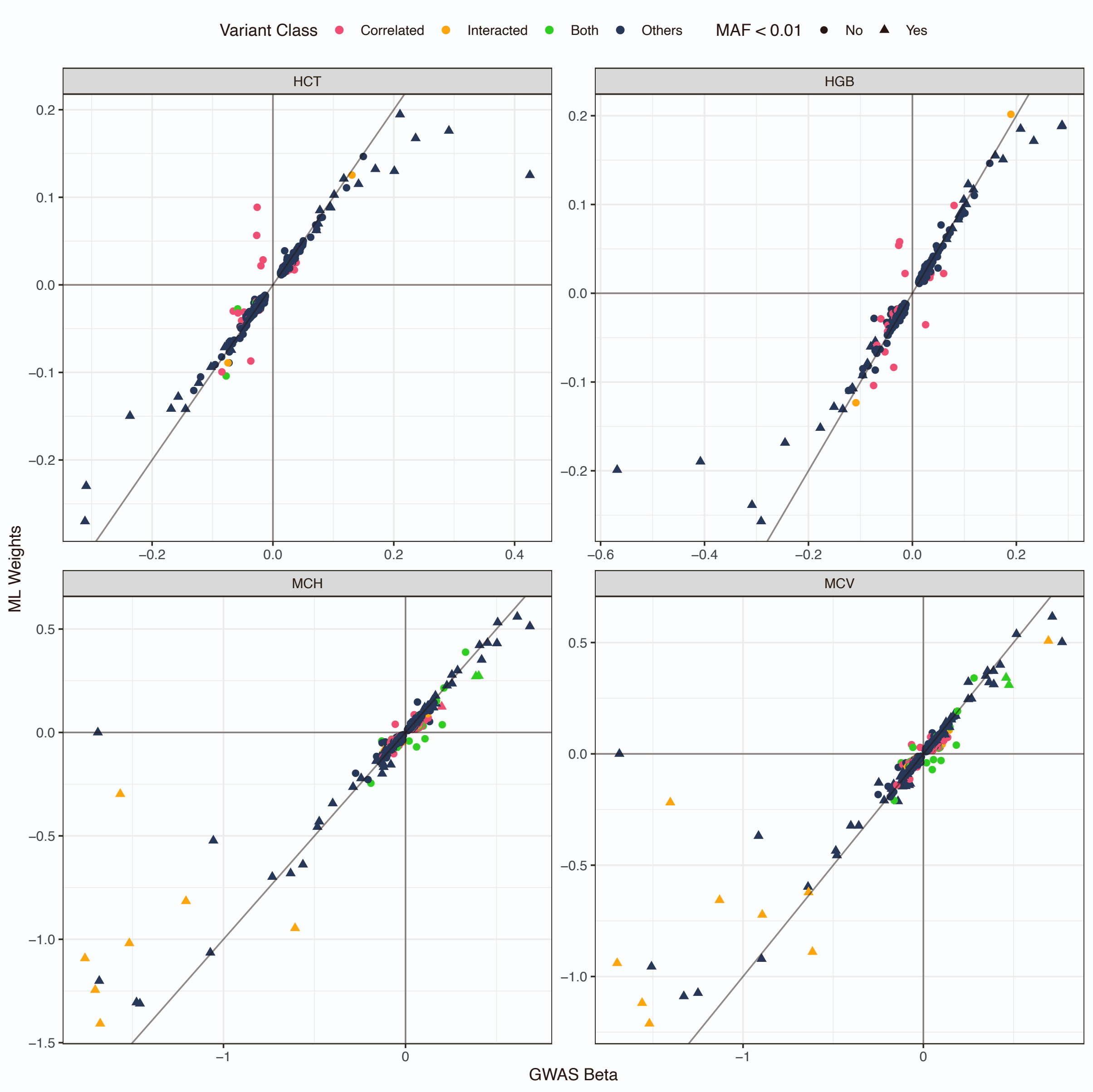

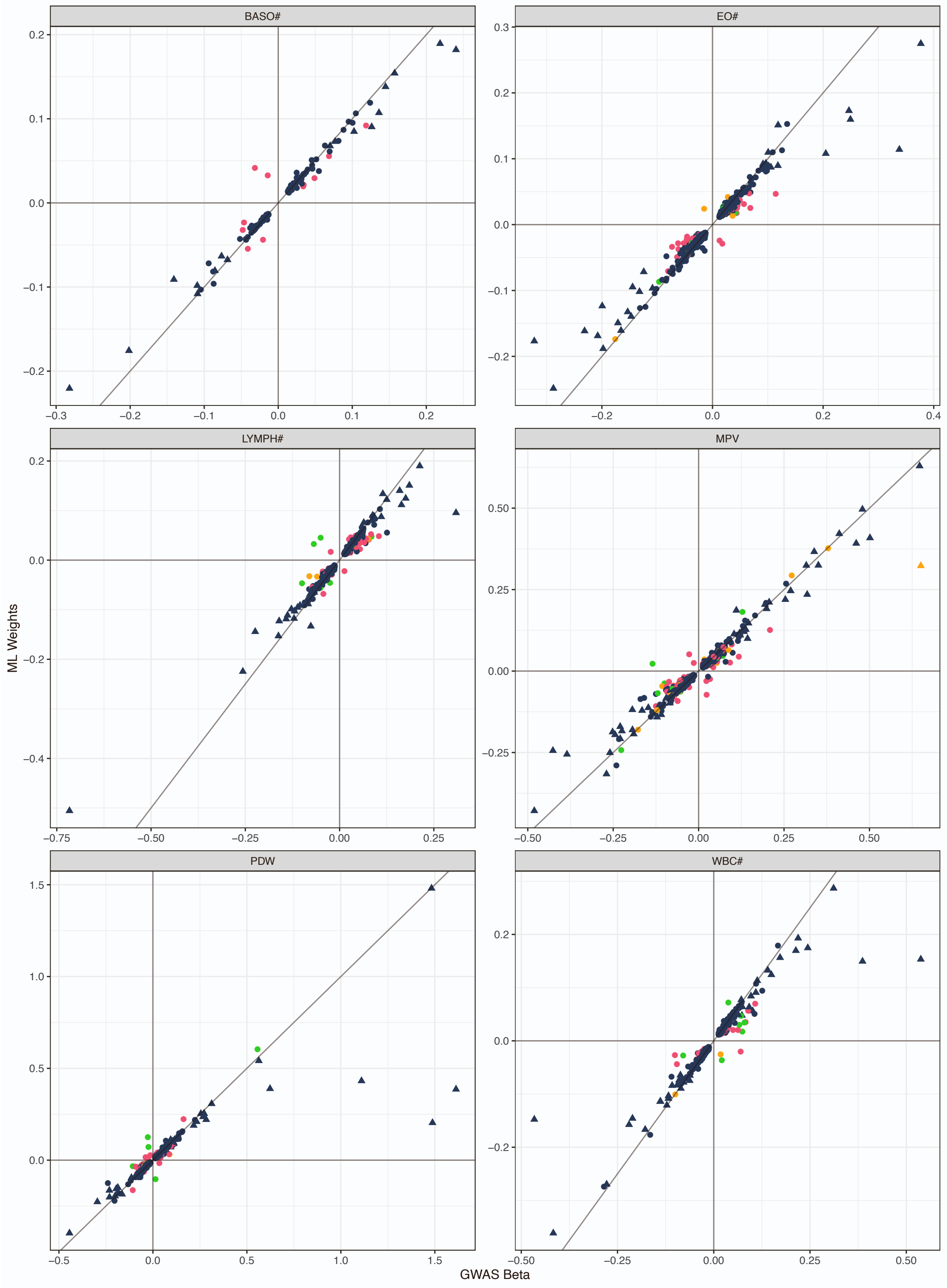
BASO#
EO#
LYMPH#
MPV
PDW
WBC#
ML Weights
GWAS Beta

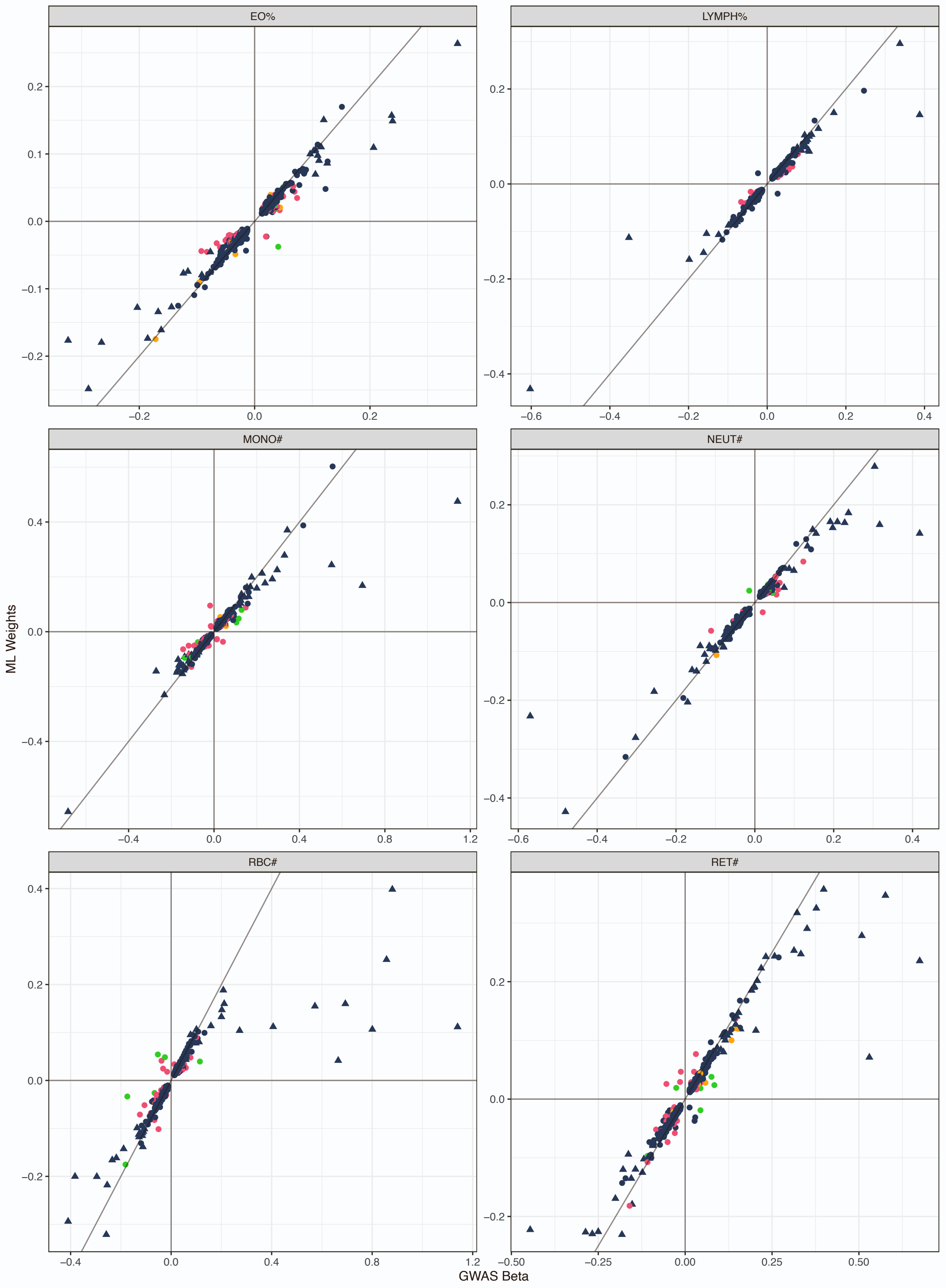
EO%
LYMPH%
MONO#
NEUT#
RBC#
RET#
ML Weights
GWAS Beta

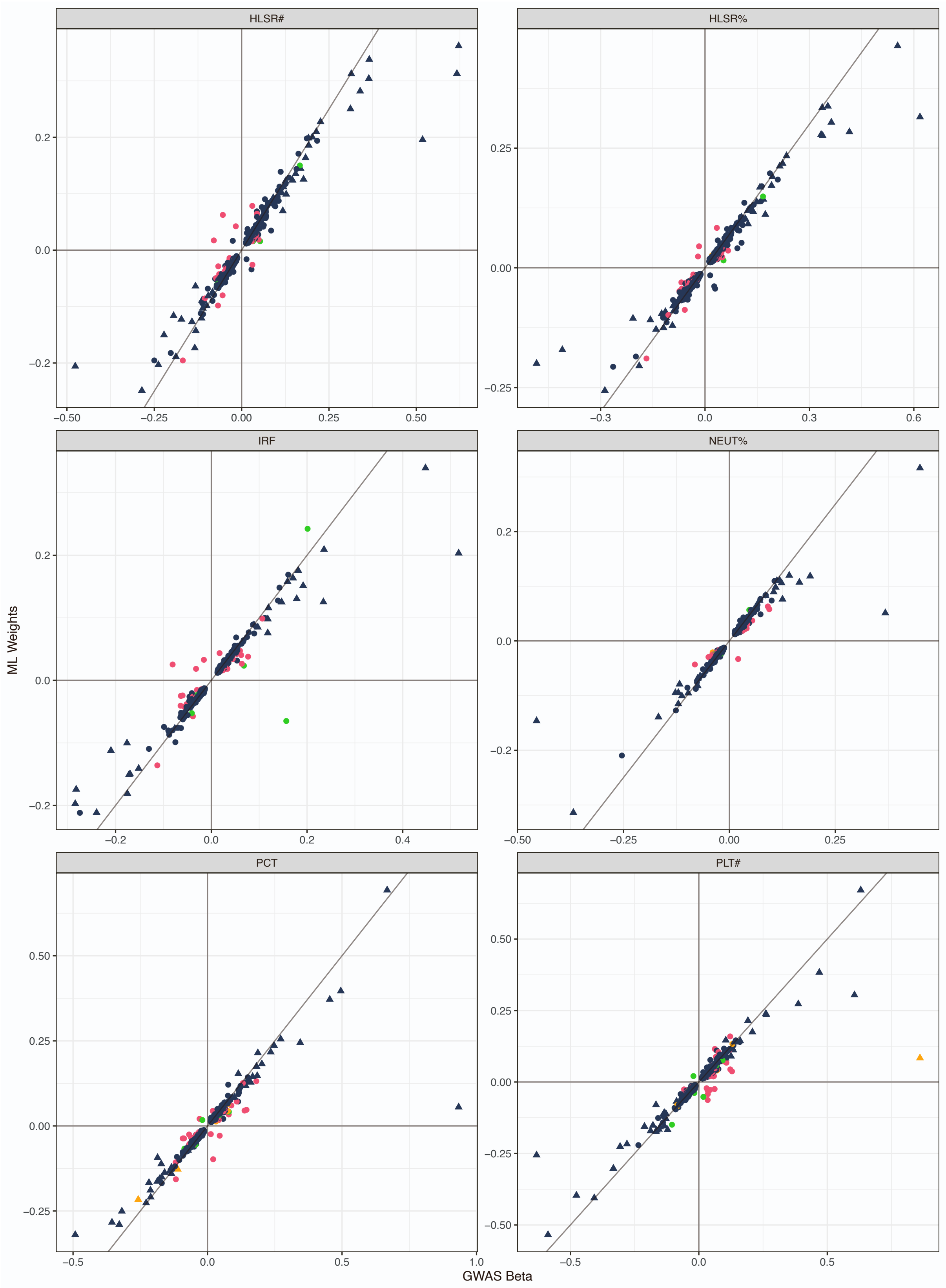
HLSR#
HLSR%
IRF
NEUT%
PCT
PLT#
ML Weights
GWAS Beta

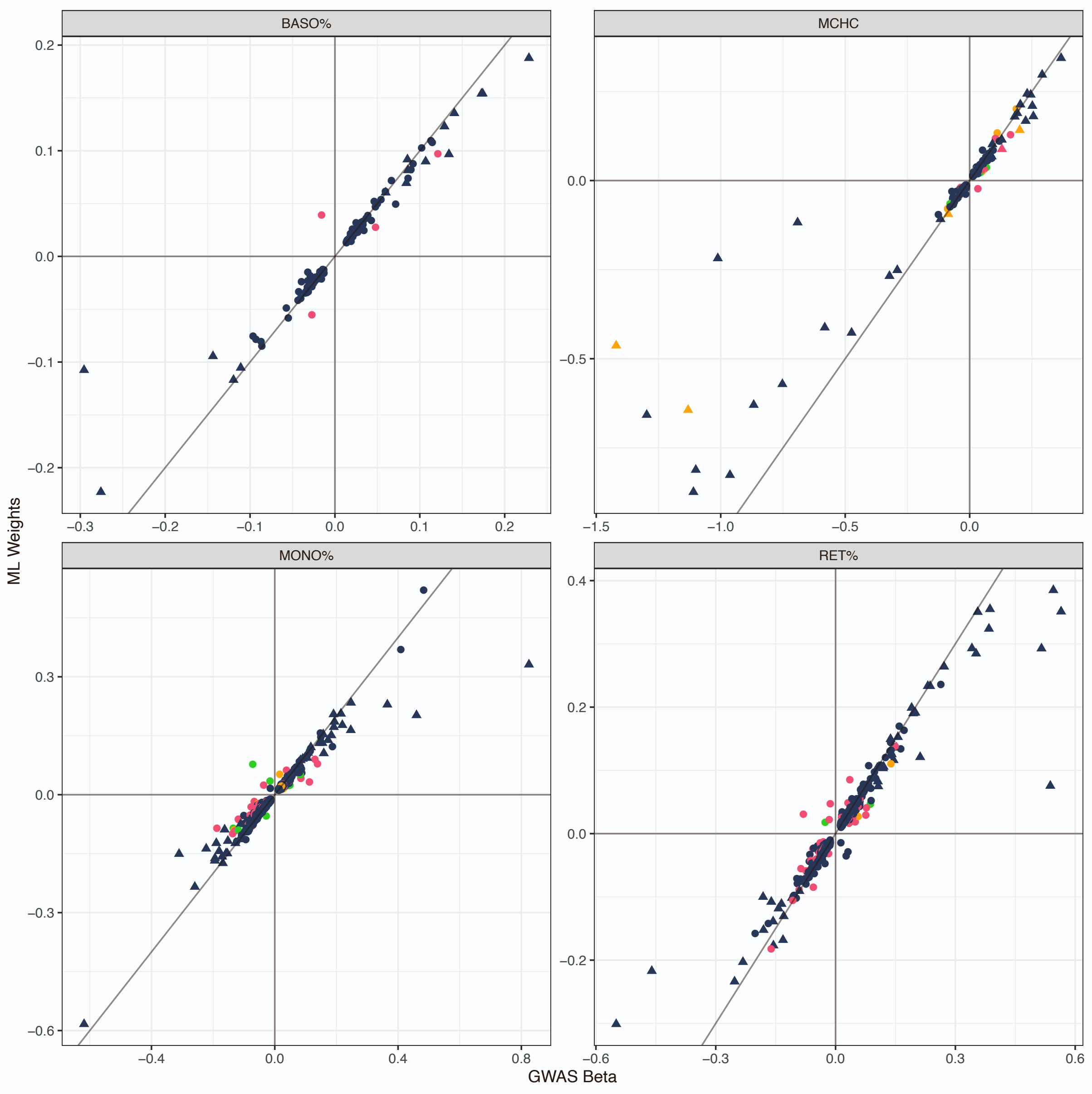

BASO%
MCHC
MONO%
RET%
ML Weights
GWAS Beta
0.2
0.1
0.0
−0.1
−0.2
−0.3
0.0
−0.5
−1.5
−1.0
0.3
−0.3
−0.6
−0.4
0.4
0.8
0.6

Figure S2. Performance of P+T, EN and LDpred2 methods on different variant sets in UKB, related to Figure 3.

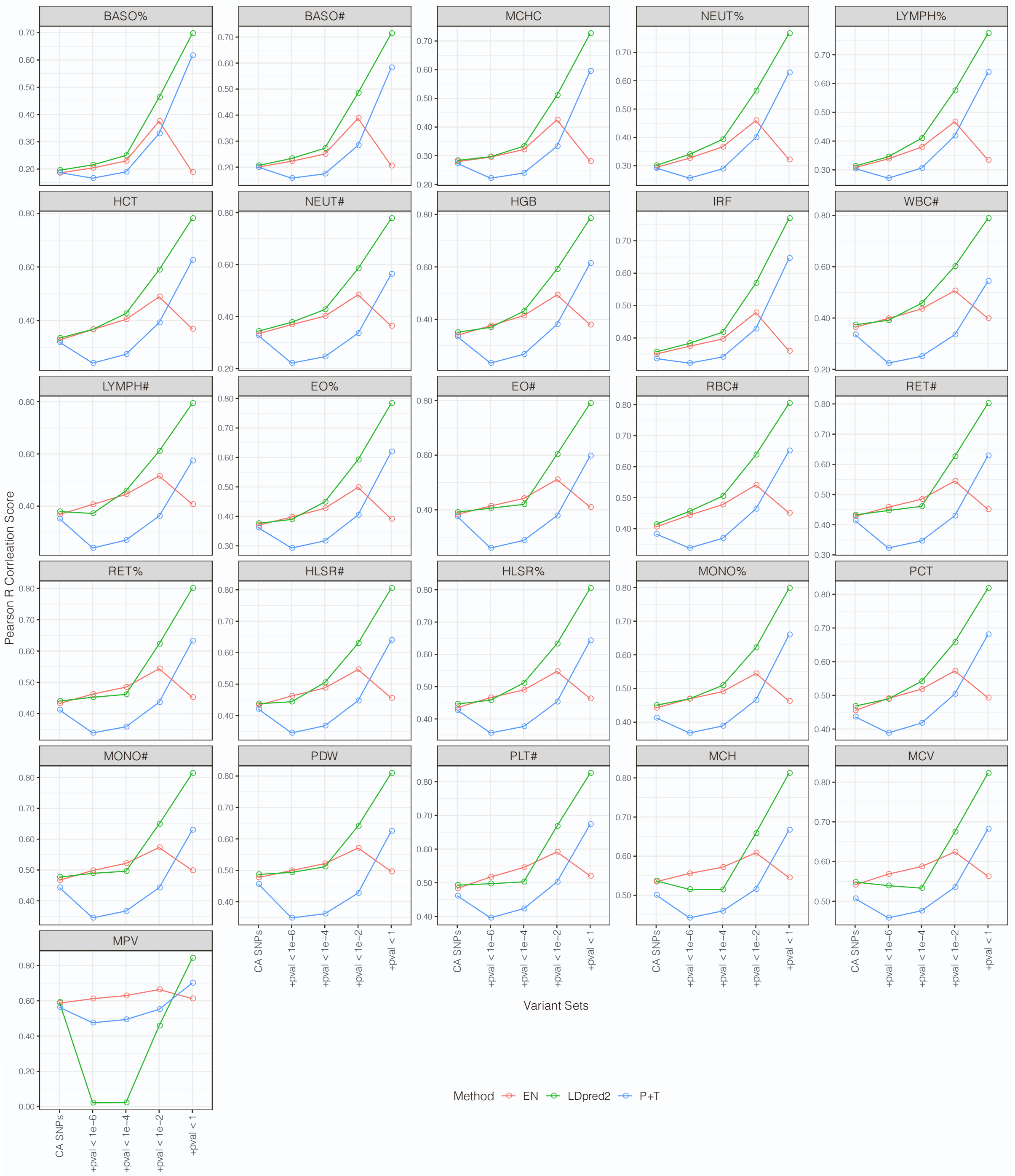

Figure S3. Trait levels by quintiles of EN-trained trait PGSs in men and women for the other 23 blood cell traits on INTERVAL, related to Figure 4. The traits are ordered by their PGS *r* scores (trained using EN on the largest variant set) in INTERVAL.

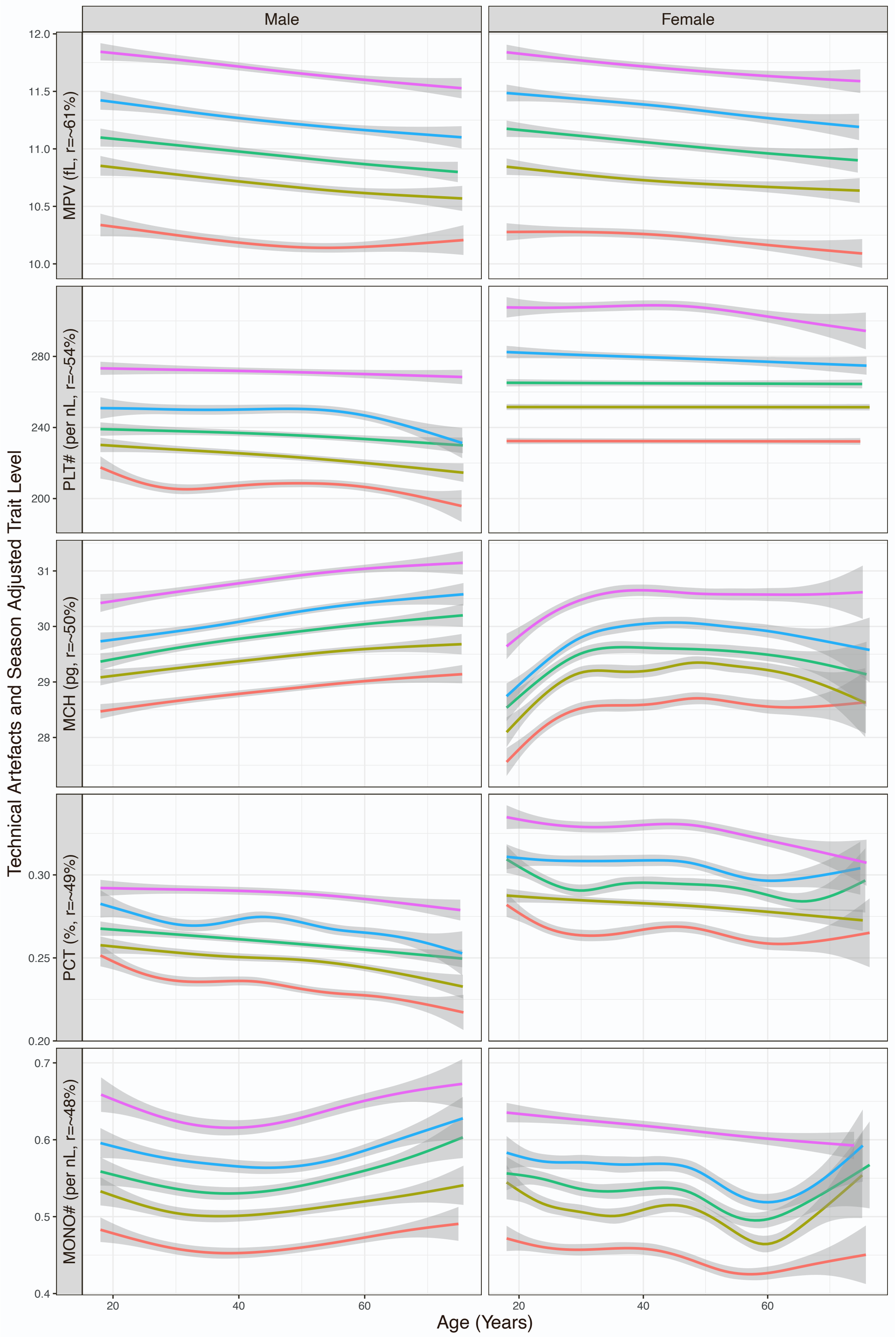

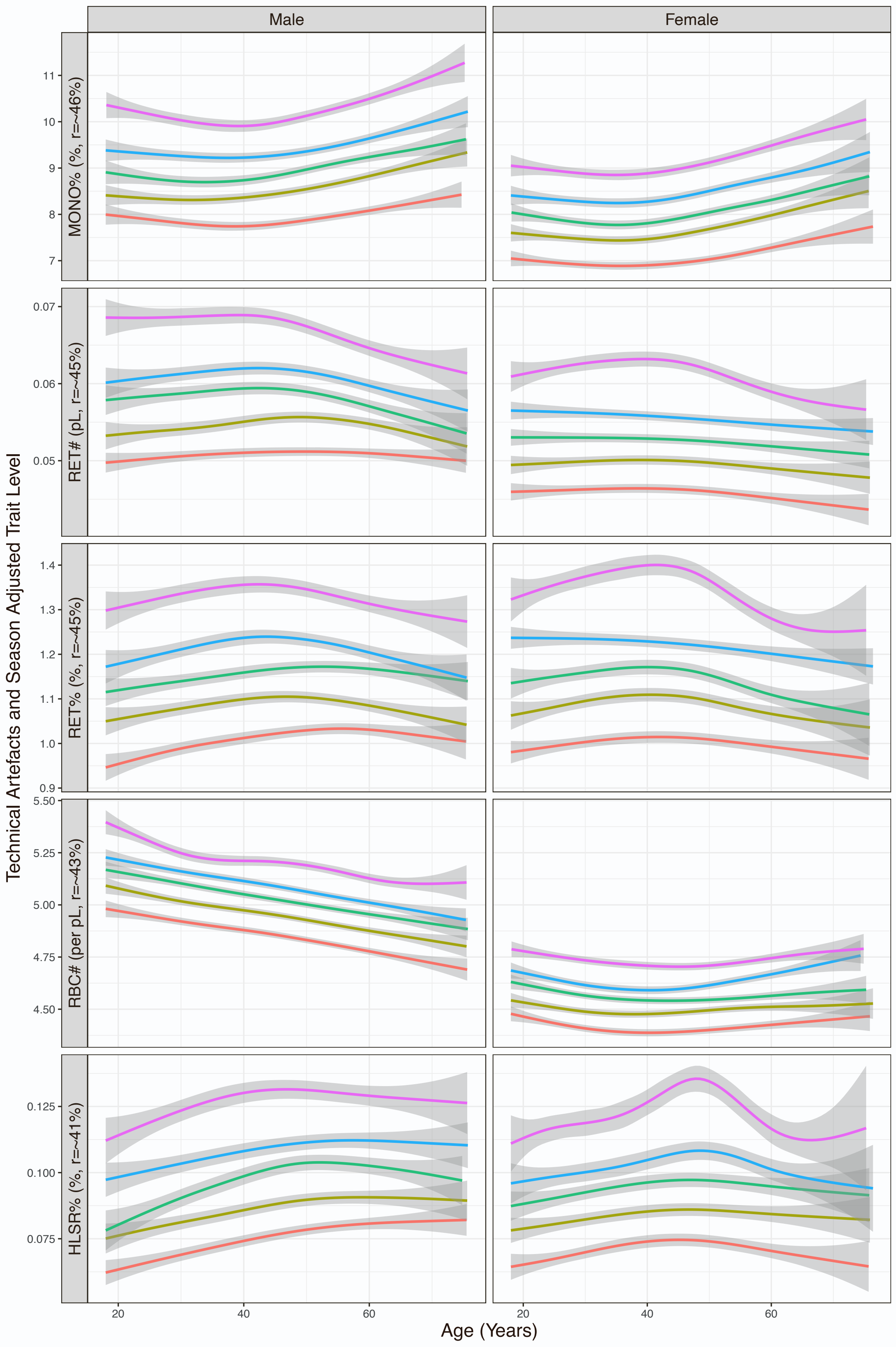

Male
Female
MONO% (%, r=~46%)
RET# (pL, r=~45%)
RET% (%, r=~45%)
RBC# (per pL, r=~43%)
HLSR% (%, r=~41%)
Technical Artefacts and Season Adjusted Trait Level
Age (Years)

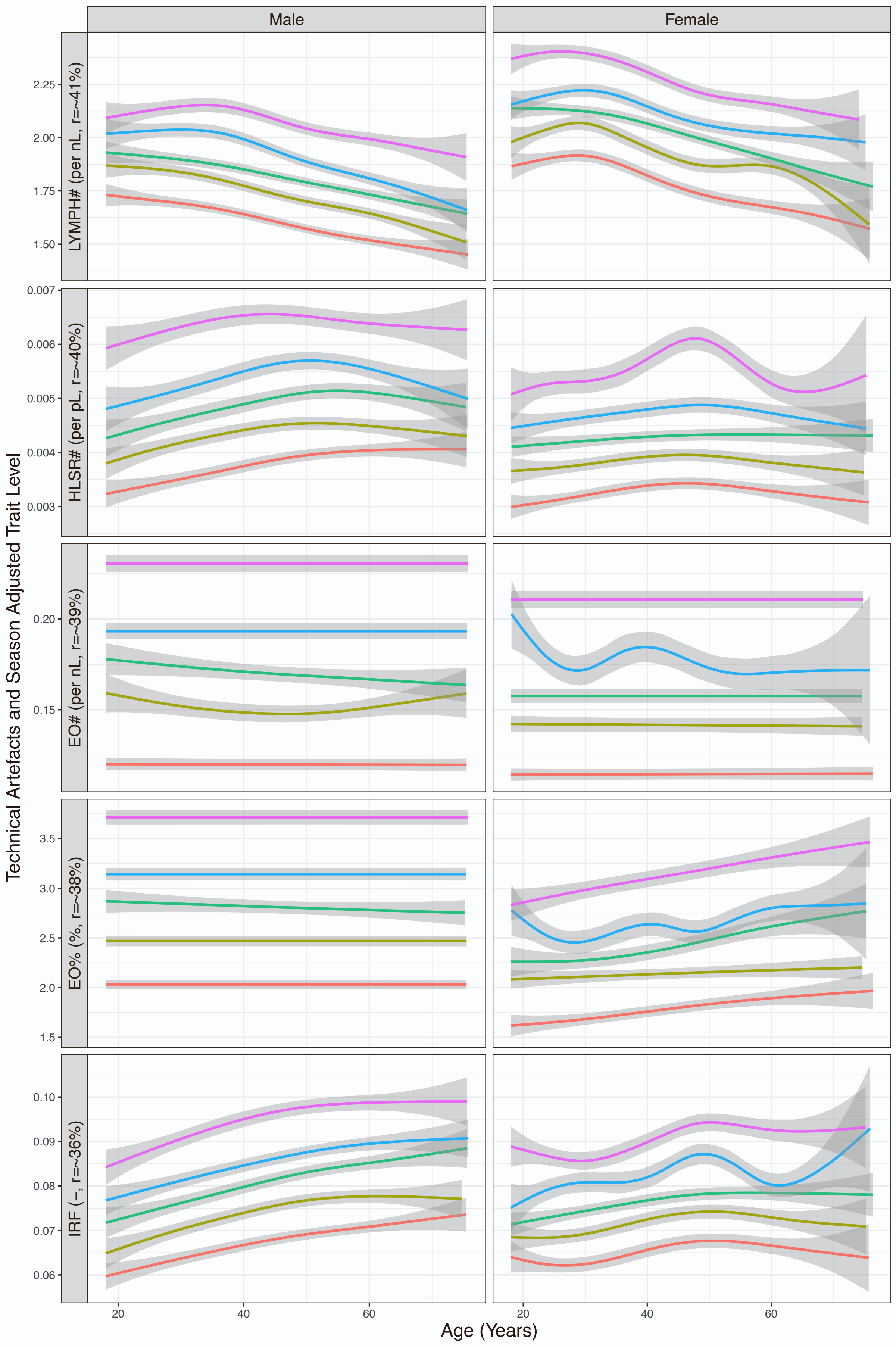
Male
Female
LYMPH# (per nL, r=~41%)
HLSR# (per pL, r=~40%)
EO# (per nL, r=~39%)
EO% (%, r=~38%)
IRF (−, r=~36%)
Technical Artefacts and Season Adjusted Trait Level
Age (Years)
2.25
2.00
1.75
1.50
0.007
0.006
0.005
0.004
0.003
0.20
0.15
3.5
3.0
2.5
2.0
1.5
0.10
0.09
0.08
0.07
0.06
20
40
60

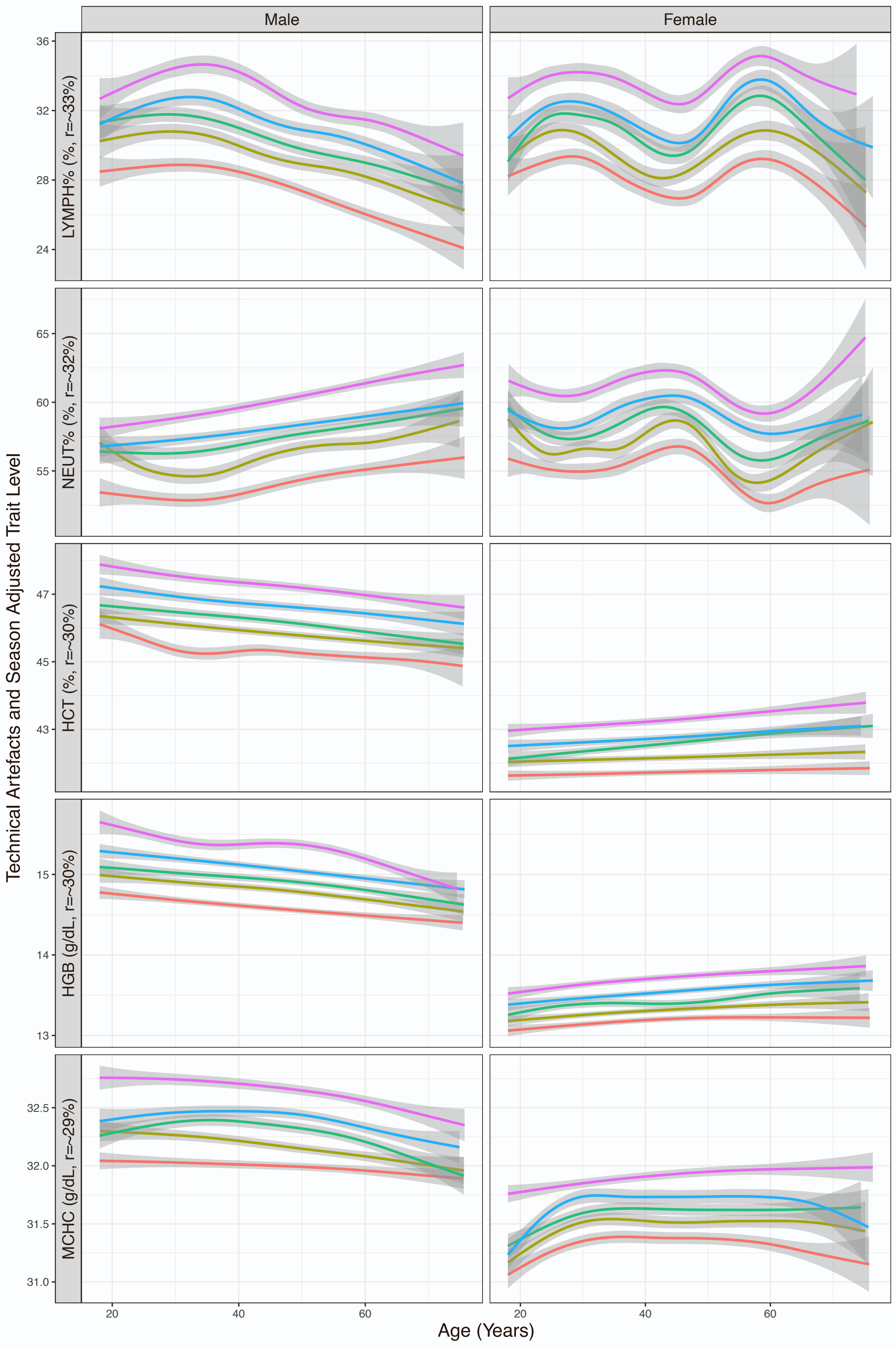

Male
Female
Technical Artefacts and Season Adjusted Trait Level
LYMPH% (%, r=~33%)
NEUT% (%, r=~32%)
HCT (%, r=~30%)
HGB (g/dL, r=~30%)
MCHC (g/dL, r=~29%)
Age (Years)

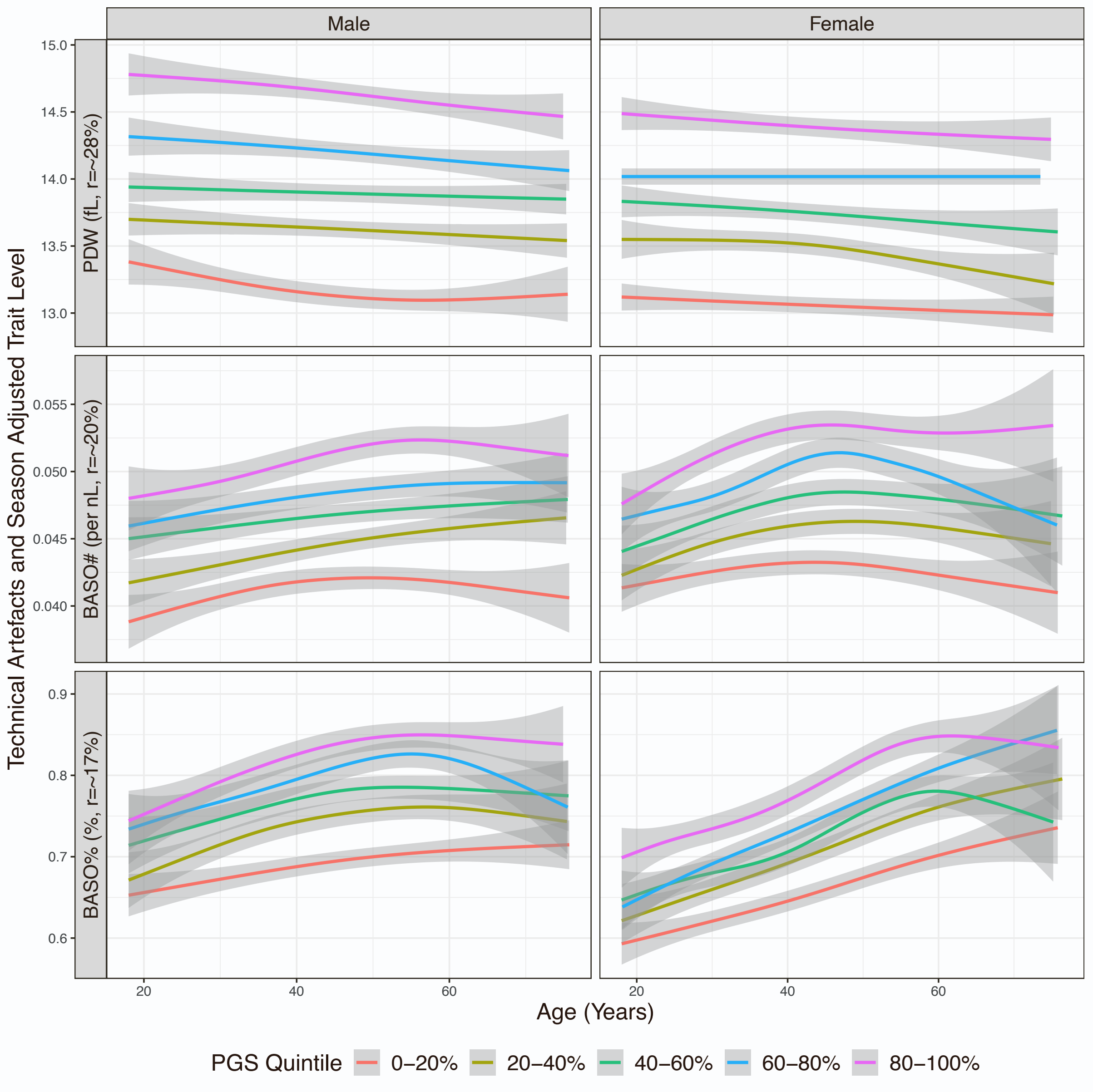

Male
Female
Technical Artefacts and Season Adjusted Trait Level
PDW (fL, r=~28%)
BASO# (per nL, r=~20%)
BASO% (%, r=~17%)
15.0
14.5
14.0
13.5
13.0
0.055
0.050
0.045
0.040
0.9
0.8
0.7
0.6
20
40
60
Age (Years)
PGS Quintile
0–20%
20–40%
40–60%
60–80%
80–100%

Figure S4. An example of a three-layer MLP, related to STAR Methods. The output $y = f^3(f^1(SNP_1, SNP_2, SNP_3)\cdot f^2(SNP_1, SNP_2, SNP_3))$

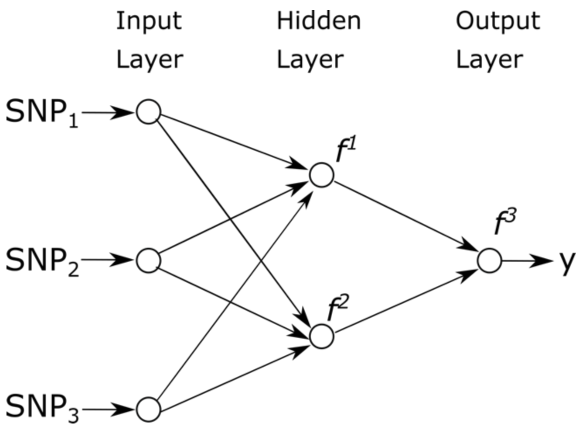

Figure S5. (a) An example of a one-dimensional CNN. (b) An example of a convolution operation. (c) An example of a max pooling operation, related to STAR Methods. The convolution kernel in (b) has a size of 1*2 and operates with a stride of 1. The max pooling filter in (c) has a size 1*2 and operates with a stride of 1. The CNN in (a) has an input of a one-dimensional vector with *n* units, and has a convolution layer and a pooling layer. The dimension *m* of a newly generated representation via a convolution operation relies on the size of the kernel being applied as well as other possible factors, e.g. padding approaches, and the number of new representations *l* is equivalent to the number of kernels used in the model. The dimension *k* of a new representation after pooling is decided by the filter size being used.

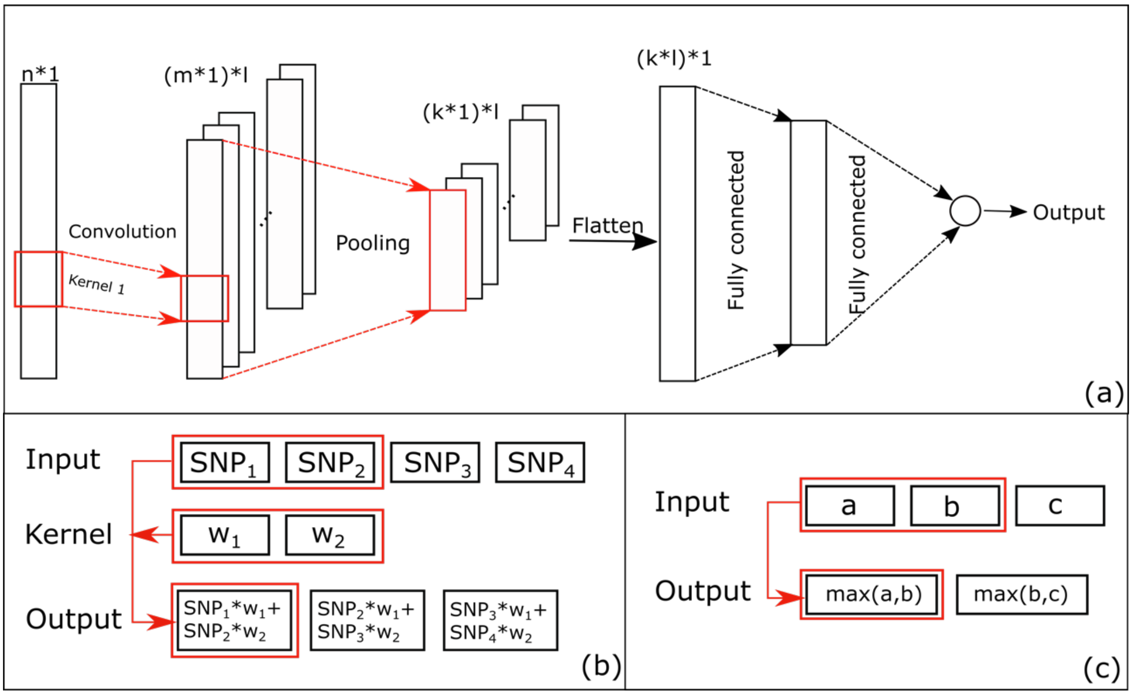

Supplement: Document S1. Tables S1 and S2 and Figures S1–S5 [file mmc1.pdf]
