# Supplementary material for: Machine learning optimized polygenic scores for blood cell traits identify sex-specific trajectories and genetic correlations with disease
Source: Cell Genom. 2022 Jan 13;2(1):100086. doi: 10.1016/j.xgen.2021.100086 (PMC8758502; doi:10.1016/j.xgen.2021.100086)

Cell Genomics

Article

Machine learning optimized polygenic scores for blood cell traits identify sex-specific trajectories and genetic correlations with disease

Graphical abstract

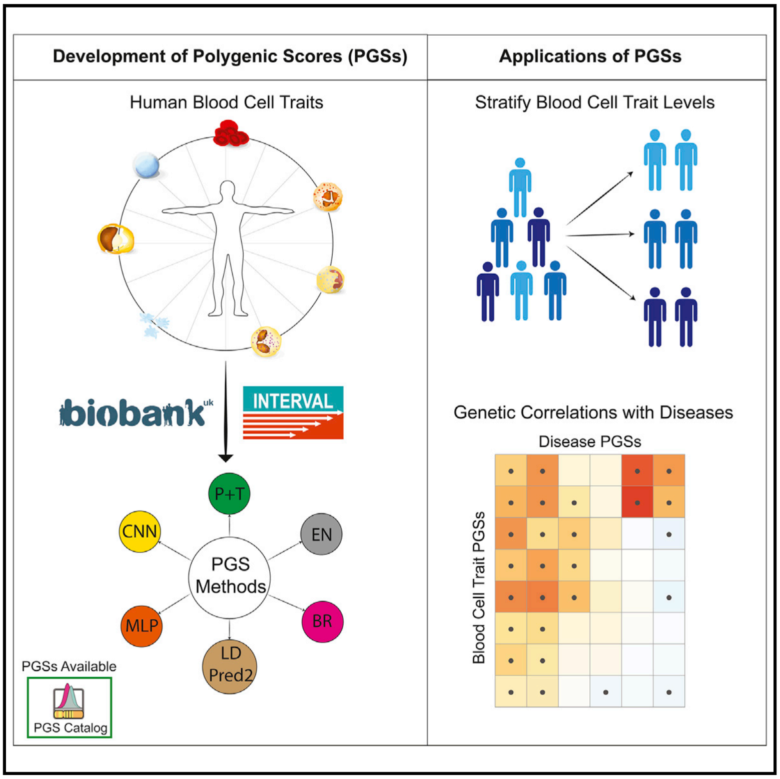

Authors

Yu Xu, Dragana Vuckovic, Scott C. Ritchie, ..., John Danesh, Nicole Soranzo, Michael Inouye

Correspondence

yx322@medschl.cam.ac.uk (Y.X.), mi336@medschl.cam.ac.uk (M.I.)

In brief

Xu et al. develop and validate polygenic scores (PGSs) for 26 blood cell traits using 6 PGS methods. PGSs developed using machine learning methods show improved polygenic prediction and allow for jointly modeling the effect of correlation, interaction, and low MAF variants. Blood cell trait PGSs were used to stratify the age-based trajectories of blood cell trait levels and showed genetic correlation with common diseases.

Highlights

- Evaluated the performance of 6 polygenic scoring methods
- Developed machine learning-optimized PGSs for blood cell traits
- PGSs showed interaction with sex and stratify age-dependent blood cell trait levels
- PGSs of blood cell traits were genetically correlated with common diseases

Xu et al., 2022, Cell Genomics 2, 100086
January 12, 2022 © 2021 The Author(s).
https://doi.org/10.1016/j.xgen.2021.100086

CellPress

Cell Genomics

CellPress
OPEN ACCESS

Article

Machine learning optimized polygenic scores for blood cell traits identify sex-specific trajectories and genetic correlations with disease

Yu Xu,[1,2,3,14,*] Dragana Vuckovic,[4,5] Scott C. Ritchie,[1,2,3,6] Parsa Akbari,[3,5] Tao Jiang,[3] Jason Grealey,[2,7] Adam S. Butterworth,[3,5,6,12] Willem H. Ouwehand,[4,6,8,9] David J. Roberts,[5,8,10] Emanuele Di Angelantonio,[3,5,6,11,12] John Danesh,[3,4,5,6,12] Nicole Soranzo,[4,5,6] and Michael Inouye[1,2,3,6,12,13,*]

[1]Cambridge Baker Systems Genomics Initiative, Department of Public Health and Primary Care, University of Cambridge, Cambridge CB1 8RN, UK
[2]Cambridge Baker Systems Genomics Initiative, Baker Heart and Diabetes Institute, Melbourne, VIC 3004, Australia
[3]British Heart Foundation Cardiovascular Epidemiology Unit, Department of Public Health and Primary Care, University of Cambridge, Cambridge CB1 8RN, UK
[4]Department of Human Genetics, Wellcome Sanger Institute, Hinxton CB10 1SA, UK
[5]National Institute for Health Research Blood and Transplant Research Unit in Donor Health and Genomics, University of Cambridge, Cambridge CB1 8RN, UK
[6]British Heart Foundation Centre of Research Excellence, University of Cambridge, Cambridge CB1 8RN, UK
[7]Department of Mathematics and Statistics, La Trobe University, Bundoora, VIC 3086, Australia
[8]National Health Service (NHS) Blood and Transplant, Cambridge Biomedical Campus, Cambridge CB2 0PT, UK
[9]Department of Haematology, University of Cambridge, Cambridge CB2 0PT, UK
[10]National Institute for Health Research Oxford Biomedical Research Centre, University of Oxford and John Radcliffe Hospital, Oxford OX3 9DU, UK
[11]Health Data Science Research Centre, Human Technopole, Milan 20157, Italy
[12]Health Data Research UK Cambridge, Wellcome Genome Campus and University of Cambridge, Cambridge CB10 1SA, UK
[13]The Alan Turing Institute, London NW1 2DB, UK
[14]Lead contact
*Correspondence: yx322@medschl.cam.ac.uk (Y.X.), mi336@medschl.cam.ac.uk (M.I.)
https://doi.org/10.1016/j.xgen.2021.100086

SUMMARY

Genetic association studies for blood cell traits, which are key indicators of health and immune function, have identified several hundred associations and defined a complex polygenic architecture. Polygenic scores (PGSs) for blood cell traits have potential clinical utility in disease risk prediction and prevention, but designing PGS remains challenging and the optimal methods are unclear. To address this, we evaluated the relative performance of 6 methods to develop PGS for 26 blood cell traits, including a standard method of pruning and thresholding (P + T) and 5 learning methods: LDpred2, elastic net (EN), Bayesian ridge (BR), multilayer perceptron (MLP) and convolutional neural network (CNN). We evaluated these optimized PGSs on blood cell trait data from UK Biobank and INTERVAL. We find that PGSs designed using common machine learning methods EN and BR show improved prediction of blood cell traits and consistently outperform other methods. Our analyses suggest EN/BR as the top choices for PGS construction, showing improved performance for 25 blood cell traits in the external validation, with correlations with the directly measured traits increasing by 10%–23%. Ten PGSs showed significant statistical interaction with sex, and sex-specific PGS stratification showed that all of them had substantial variation in the trajectories of blood cell traits with age. Genetic correlations between the PGSs for blood cell traits and common human diseases identified well-known as well as new associations. We develop machine learning-optimized PGS for blood cell traits, demonstrate their relationships with sex, age, and disease, and make these publicly available as a resource.

INTRODUCTION

Blood cells play essential roles in a variety of biological processes, such as oxygen transport, iron homeostasis, and pathogen clearance.[1–3] Abnormalities in blood cell traits, such as the number of cells, the proportions of different types, sizes, and morphology, and thus their likely functions, have been associated with a range of human diseases, such as reticulocyte indices with coronary heart disease[4] or eosinophil counts with asthma.[5] As such, blood cell counts and associated traits are also widely used in clinical practice, where they are among the most common clinical tests worldwide.

This is an open access article under the CC BY license (http://creativecommons.org/licenses/by/4.0/).

Blood cell traits are heritable, and their genetic architecture has been found to be polygenic. Analyses of the UK Biobank (UKB)[6,7] and INTERVAL[8] cohorts have suggested that between 18% and 30% of the variance in erythrocyte counts and morphology can be explained by hundreds of common autosomal variants.[4] It is expected, therefore, that levels of these traits can, to some extent, be predicted by genetic variation through the use of polygenic scores (PGSs)[9].

PGSs for blood cell traits show the potential for utility in clinical practice. A recent study examining the effects of known pathogenic variants and blood cell trait PGSs on patients with rare blood disorders showed that a 1-standard deviation (SD) increase in PGS was comparable in risk to carrying a rare coding variant in heterozygosity.[10] These results indicate that PGSs for blood cell traits could play important roles in disease risk prediction and prevention, or help in better understanding disease etiology and identifying novel therapeutic targets.[11,12]

A PGS is most commonly constructed as a weighted sum of genetic variants, typically single-nucleotide polymorphisms (SNPs), carried by an individual, in which the genetic variants are selected and their weights are set via the per-SNP univariate analysis in a genome-wide association study (GWAS).[9,13] Univariate analysis largely relies on hard cutoff thresholds to identify associated variants—for example, linkage disequilibrium (LD) pruning for selection of independent variants[14] and p value thresholding for selection of significant variants (the P + T method). However, standard methods such as P + T have limitations, including that they do not capture interactions between variants. Machine learning and deep learning methods may provide significantly improved polygenic scores for blood cell traits, as has been demonstrated in applications for celiac disease and type 1 diabetes,[15–19] thus facilitating analyses into the genetic architecture of blood cell traits and their relationships with sex- and age-specific effects and the genetics of common diseases.

In this study, we evaluate 6 PGS methods to develop optimized PGS for 26 blood cell traits across 3 blood cell types—platelets, red blood cells, and white blood cells—using data from UK Biobank and INTERVAL (see Figure 1 for study workflow). The 6 PGS methods evaluated in this study include the pruning and thresholding (P + T) method and 5 learning methods: LDpred2, elastic net (EN), Bayesian ridge (BR), multilayer preceptron (MLP), and convolutional neural network (CNN). Our analysis finds that common machine learning methods EN and BR show improved polygenic prediction of blood cell traits and consistently outperform other methods. We assess the compositions of these blood cell trait PGSs and discover that the benefits of EN and BR are in jointly modeling the effect of correlation, interaction, and low minor allele frequency (MAF) variants. Our analyses suggest that the EN and BR methods are the top choices for PGS construction of blood cell traits when sufficient individual-level data are available. When there is no sufficient individual-level data available, LDpred2 is also a good option. We investigate the interactions of PGS with sex as well as stratification of measured blood cell traits across ages. Finally, we perform a genetic correlation scan of blood cell trait PGSs across diverse common diseases. We make the machine learning-optimized PGS models publicly available via the PGS Catalog[20] to facilitate genetic and clinical studies on blood cell traits and associated diseases.

RESULTS

Development of blood cell trait PGSs

Using the optimal variant set (i.e., conditional analysis variants, see STAR Methods) identified by the P + T method, we compared the performance of the 5 learning methods with that of P + T for constructing PGSs for 26 blood cell traits (Figure 2). Four of the 5 methods, EN, BR, LDpred2, and MLP, consistently outperform the P + T method in terms of Pearson *r* for nearly every blood cell trait. Notably, the performance of EN and BR were nearly indistinguishable and were the most stable as well as the top-performing methods overall. Although LDpred2 outperformed other learning methods in the internal validation across the majority of the traits, its outperformance largely declined in the external validation with similar or slightly better performance for most traits and notable underperformance for a few traits when compared with EN and BR (e.g., basophil percentage of white cells [BASO%]). With any of these 4 learning methods, PGSs for 11 blood cell traits achieved a nearly ≥ 0.02 increase in Pearson *r* score in internal validation. The following 5 blood cell traits each achieved ≥ 0.02 improvement in both internal and external validation using EN or BR, in comparison with the P + T method (monocyte percentage [MONO%], white blood cell count [WBC#], mean platelet volume [MPV], monocyte count [MONO#] and plateletcrit [PCT) (Figure 2). We found that the incorporation of nonlinear factors, as in MLP and CNN, did not improve genomic prediction of blood cell traits, compared with linear models. For nearly half of the blood cell traits we studied, the CNN resulted in PGS with approximately the same or lower Pearson *r* as the P + T approach.

Comparing estimated SNP effect sizes between univariate analysis and machine learning

BR and EN outperformed P + T due largely to differences in variant effect size estimation; thus, we compared the variant effect sizes estimated by univariate analysis (used in the P + T method) and the EN/BR methods (Figure S1). We found that almost no effect sizes were set to zero by BR and EN, and effect sizes of most variants using BR or EN are the same or similar to those from the univariate analysis in GWAS. This is consistent with a genetic model in which most common genetic variants are independently and additively contributing to each blood cell trait. In addition, we also found that both EN and BR tended to shrink the effects (sometimes greatly) of variants with low MAF compared with that estimated in univariate analysis; however, this did not necessarily contribute to substantially improved PGSs. For example, we observed the effects of numerous low-MAF variants for traits such as mean corpuscular volume (MCV) and mean corpuscular hemoglobin concentration (MCHC) were substantially shrunk by BR and EN; PGS construction of MCV achieved significant improvement ($\sim$0.03 increase in Pearson *r* score), while PGS for MCHC saw little improvement in internal validation (Figure 2). In spite of that, the effect of shrinkage of low-MAF variants can result in better model generalization, which means they can offer more stable predictions when applied across datasets. This is likely due to the substantial noise in univariate

CellPress
OPEN ACCESS

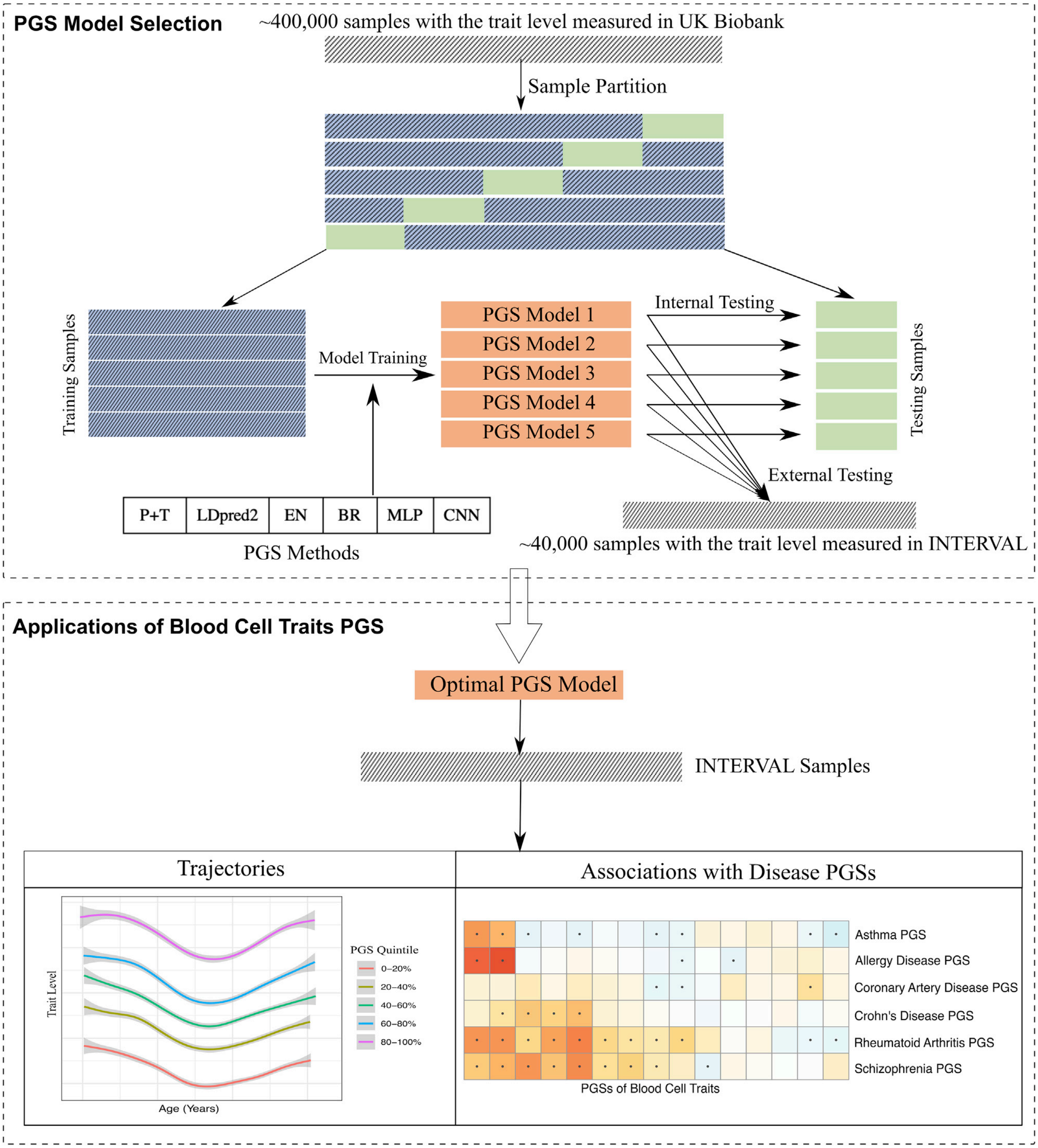

Figure 1. PGS construction of blood cell traits using 6 different methods

Six PGS methods were evaluated in this study: pruning and thresholding (P + T) and 5 learning methods: LDpred2, elastic net (EN), Bayesian ridge (BR), multilayer preceptron (MLP), and convolutional neural network (CNN).

estimates for effect sizes of low-MAF variants in existing training samples.

Univariate analysis does not consider LD among variants, which is a well-known cause for reduced PGS accuracy. The P + T method relies on LD pruning to remove correlations among variants; however, it must make trade-offs between removing correlated variants and keeping predictive variants by using hard cutoff thresholds. The selected conditional analysis (CA)

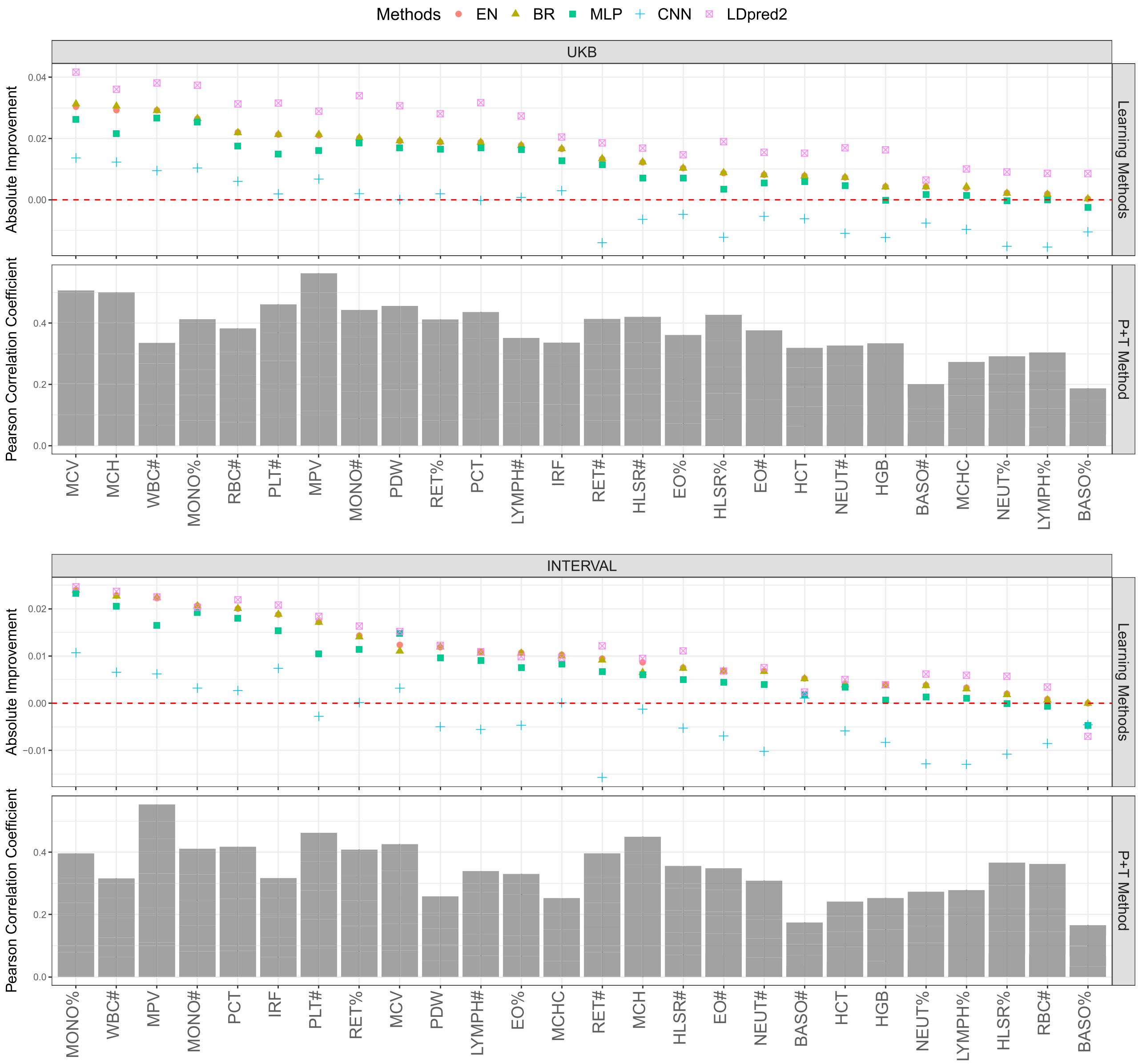

Figure 2. Performance comparison of 5 learning methods with the P + T method

Pearson *r* score performance of the P + T method for PGS construction of 26 blood cell traits are presented in testing on UKB or INTERVAL. Relative to the P + T method, performance of the 5 learning methods: EN, BR, LDpred2, MLP, and CNN, are presented for each blood cell trait in descending order, left to right, according to EN (largest Pearson *r* increases on left). Given a particular method, a trait and a cohort, the averaged *r* performance of the 5 trained models, corresponding to the 5 different training-testing data partitions, is shown.

Detailed comparison between variant effect sizes estimated using EN/BR and P + T are presented in Figure S1.

variants for each trait included many correlated variants, with those with $r^2 > 0.1$. As expected, for variants in moderate to high LD, EN and BR tended to assign weights that were more different from univariate analysis than for variants not in LD, with some variant effects even changing direction (Figure S1).

In addition, univariate analysis does not model SNP-SNP interaction effects on the trait; however, modeling interactions can improve PGS construction.[21] For all CA variants, we performed a SNP-SNP interaction analysis on each trait using a Bonferroni-adjusted threshold to determine significant interactions (STAR Methods). We found that significant SNP-SNP interactions tended to include SNPs that had different weights when comparing EN/BR to univariate analysis (Figure S1)—for example, in MCV and mean corpuscular hemoglobin (MCH). The increased performance of EN and BR relative to univariate analysis appeared to be due to the weights assigned for the 3 groups of variants above (low MAF, moderate to high LD, or SNP-SNP interactions).

EN and LDPred2 improve blood cell trait PGSs on larger variant sets

The above analysis has shown that EN, BR, and LDpred2 are the most promising methods for PGS construction of blood cell traits. We further explored potential improvements by incorporating larger variant sets for LDpred2 and EN (because EN and BR performed nearly identically, we only present results for EN). Our results showed that EN further improved PGS for almost every trait with the expanded variant sets, when compared with EN using conditional analysis variants only (Figures 3 and S2). For example, when incorporating all variants genome-wide (i.e., no p value threshold), 25 of the 26 traits had a further improvement of at least 0.02 in terms of *r* score in the external validation, in which 6 traits achieved >0.05 additional improvement. When compared with the P + T method, EN achieved greater improvements for PGSs of most blood cell traits by using the largest expanded variant set. For example, 25 of the 26 traits had $\geq$10% improvement over their *r* scores achieved using P + T, in which 2 traits had >20% improvement (i.e., hematocrit [HCT; from 0.24 to 0.30] and WBC# [from 0.32 to 0.39]).

By applying different p value thresholds on all the LD-thinned variants, we also obtained smaller variant sets for each trait. Our results suggested that it is possible that by using a smaller variant set with p value thresholding, EN can achieve performance comparable to when using the largest expanded variant set. For example, the performance differences in terms of *r* score using EN are within 0.01 for all of the traits between the smaller variant set with p value threshold = 10^{-4} and the largest variant set (INTERVAL external validation). Using a more lenient p value threshold (i.e., p value threshold = 10^{-2} in this study) can result in overfitting problems. By incorporating the variant set with a p value threshold of 10^{-2}, EN significantly outperformed that of using other variant sets in the internal validation with UKB, while it experienced a substantial performance decrease in the external validation with INTERVAL (Figures 3 and S2), with some models even underperforming the P + T method. However, the use of overly stringent thresholds (e.g., p value threshold = 10^{-6} or lower) could limit the predictive power of EN.

LDpred2 also showed improved performance for PGSs of 24 traits when using the expanded variant sets with more stringent p value thresholds (i.e., p value thresholds = 10^{-6} and 10^{-4}) as compared with using CA variants only. However, LDpred2 models showed overfitting on variant sets with lenient or no p value thresholding (i.e., p value thresholds = 10^{-2} and 1.0). Nevertheless, EN consistently outperformed LDpred2 in the external validation with INTERVAL data on almost every expanded variant set for every trait (Figure 3). For example, EN outperformed LDpred2 by >0.02 in terms of *r* score for 9 traits on the top-performing variant set (p value threshold of 10^{-4}) of both methods. In addition, LDpred2 failed to construct a PGS for the trait MPV on 2 expanded variant sets, indicating that EN may be more robust when using large variant sets.

Sex-specific interactions and PGS-stratified trajectories

Maximizing the accuracy and performance of PGS for blood cell traits raises opportunities for insights into the underlying biology, which is potentially of relevance to disease risk. We next compared the extent to which EN-trained PGS would be used to stratify the levels of blood cell traits in men and women over the age ranges of individuals in INTERVAL (Figures 4 and S3). There were a wide range of age-dependent dynamics in the levels of many blood cell traits in INTERVAL, with the EN-trained PGS (p value threshold = 1 in variant selection) offering stratification that was largely consistent with Pearson *r* of the trait (i.e., the larger the Pearson *r* PGS of the trait received, the better the PGS stratified the population). Blood cell traits exhibited well-known sex differences.[22] Interestingly, PGS for approximately half of blood cell traits resulted in different levels of stratification between men and women, with 10 blood cell traits passing the Bonferroni-adjusted significance threshold in PGS-sex interaction analyses (Table 1). For example, WBC indexes in women significantly decrease after menopause, while the level of these traits in men were relatively stable.[23] Importantly, in both men and women, the EN-trained PGS continued to stratify the trait levels even after the trait levels themselves changed. The average trait levels in the top versus the bottom PGS quintiles were substantially different. The top quintile of the PGS for WBC# had an additional ~1.5 WBCs per nanoliter (nL) on average in INTERVAL compared to the bottom quintile (an increase of ~25%); similarly, the difference between the top versus the bottom 1% PGSs for WBC# was ~2.2 WBCs per nanoliter (a 40% increase). For MCV, individuals in the top PGS quintile had red blood cells with ~5 femtoliters (fL) greater volume on average than those in the bottom PGS quintile, and these differences were maintained over all age ranges for both men and women.

Genetic correlations of blood cell traits and common diseases

Finally, we examined the landscape of genetic correlations for the EN-trained PGS of blood cell traits and PGS of several common human diseases (Figure 5). We found 67 genetic correlations passing Bonferroni adjusted significance ($p < 10^{-4}$), which are consistent with well-known associations between the blood cell traits themselves and the disease. For example, prior studies have demonstrated a strong association of asthma with eosinophil indices,[4] consistent with our analyses, which show that PGSs for eosinophil counts (EO#) and eosinophil percentages (EO%) were correlated with the asthma PGS. The strongest genetic correlation was between schizophrenia and WBC#, consistent with previous studies of the trait and schizophrenia risk.[24] Our analyses also uncovered the genetic correlations for previous trait-level observations for EO# and allergic disease[25] as well as WBC# and Crohn's disease.[26] In addition to the well-known associations between blood cell traits and common diseases, the genetic association scan also identified new associations. For example, PGS of the immature fraction of reticulocytes (IRF) was significantly associated with the coronary artery disease (CAD) PGS, which is related to a recent finding that reticulocyte levels have an ambivalent association with hypertension and atherosclerosis[27]; the PGS of MONO# was significantly associated with the schizophrenia PGS, which can be supported by the inflammation hypothesis in the pathogenesis of schizophrenia.[28] These demonstrated extensive genetic correlations for blood cell traits and rheumatoid arthritis, CAD, schizophrenia

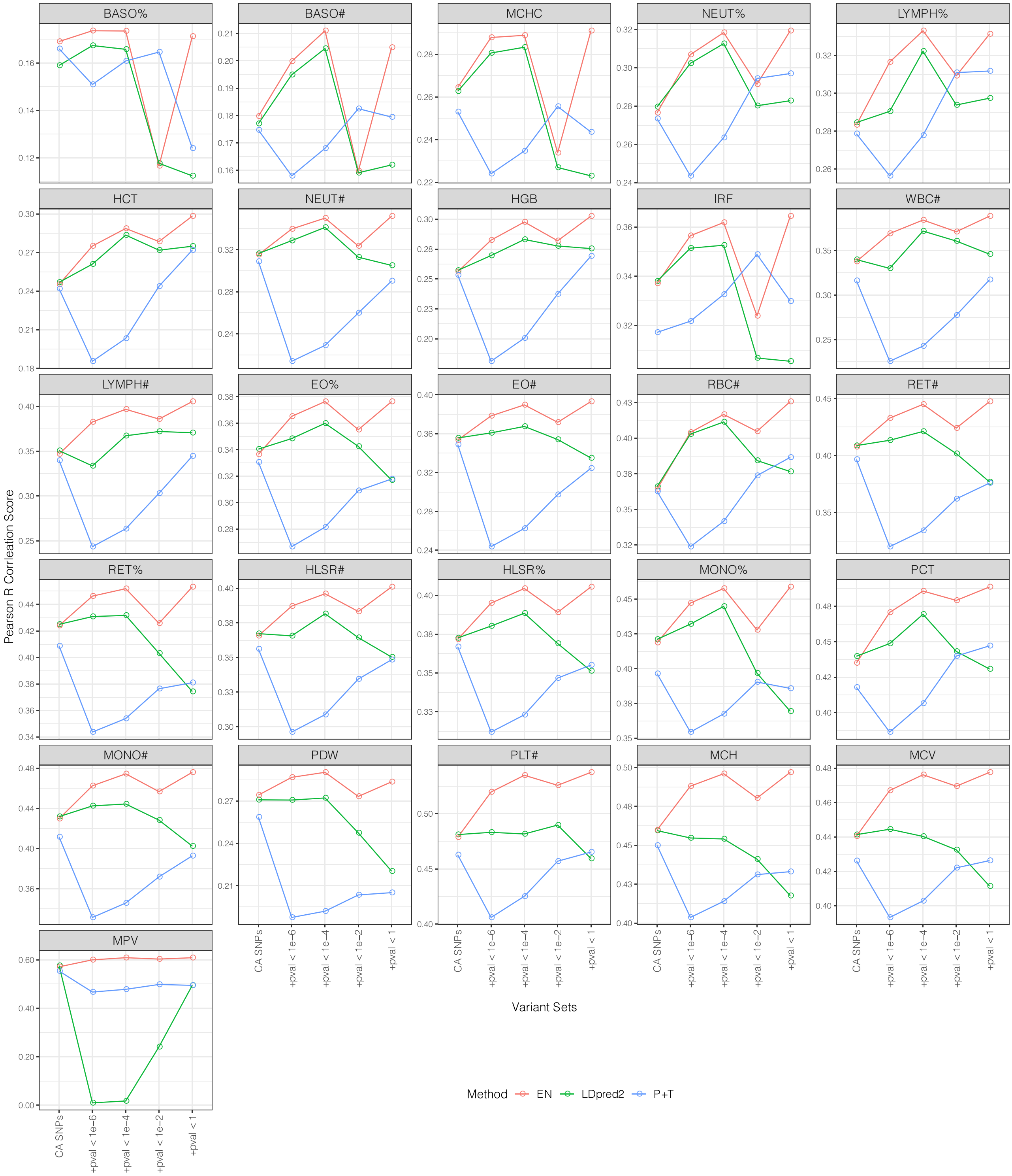

Figure 3. Performance of P + T, EN, and LDpred2 methods on different variant sets in INTERVAL

Using conditional analysis variants as a base set, we added in the selected variant sets with LD thinning and p value thresholding to form different sizes of expanded variant sets for each trait. We used the CA variant set as the starting point and then observed the performance of P + T, EN, and LDpred2 on these expanded variant sets. Note that in this figure, P + T refers to the method that directly applies the weighted sum on a given variant set with effect sizes from GWAS. See Figure S2 for similar performance comparison in UKB.

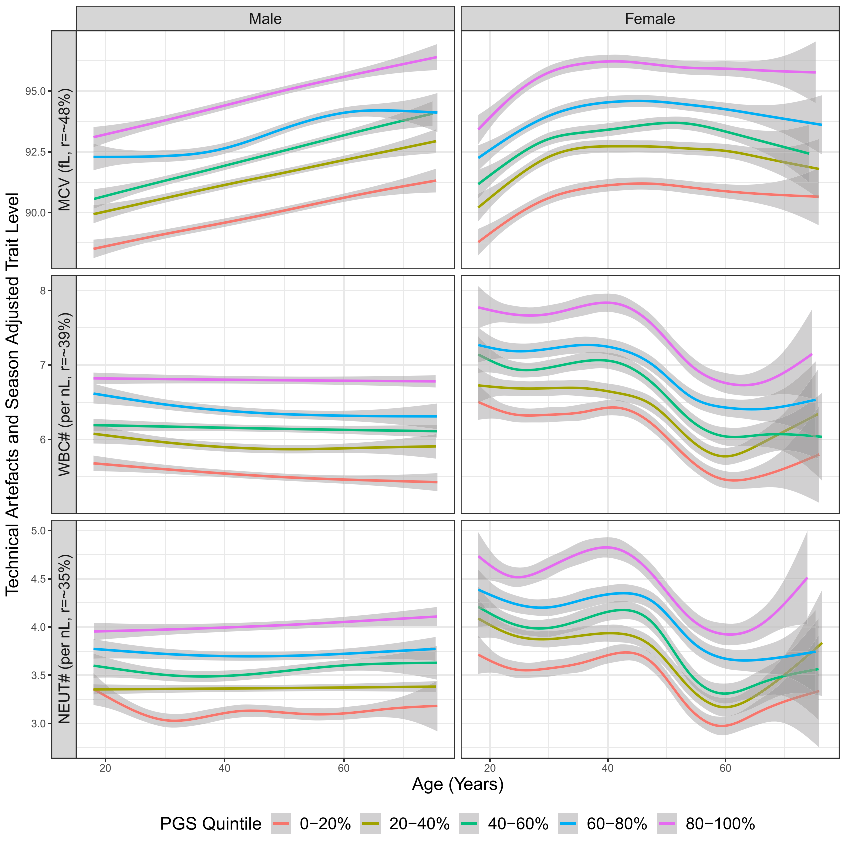

Figure 4. Trait levels by quintiles of EN-trained trait PGS in men and women for traits MCV, WBC#, and neutrophil count (NEUT#) in INTERVAL

The y axis is the observed measurements adjusted only for technical artifacts and season for each blood cell trait. The generalized additive model (GAM) was used to fit the data across INTERVAL samples, and the shaded areas represent 95% confidence intervals.

See Figure S3 for results of all other traits.

and Crohn's disease, and suggest that via shared genetics, blood cell traits may be either indicators or mediators.

DISCUSSION

Improved polygenic models of blood cells traits aid our understanding of myriad biological processes and diseases. This study demonstrated that common machine learning methods such as EN and BR show improved polygenic prediction of blood cell traits and consistently outperform other methods, likely due to their implicit modeling of SNP-SNP correlations, interactions, and controlling the effect of underrepresented low-MAF variants. We showed that blood cell trait PGSs are able to stratify age-dependent trait levels in both men and women in a population-based setting, and that many blood cell trait PGSs have sex-specific interactions. The landscape of genetic correlations between blood cell traits and common diseases identified well-known trait-level associations, such as eosinophils and asthma, and intriguing associations such as IRF and CAD, MONO# and schizophrenia.

Our analysis indicated that EN and BR can jointly model the effect of correlation, interaction, and low MAF variants, and result in improved PGSs over P + T for blood cell traits. To overcome the scalability problem in PGS methods such as MLP and CNN and provide directly comparable results across these methods, the comparative analysis was conducted based on the optimal variant sets identified by the P + T method. Due to the use of hard cut-off thresholds in P + T, this unified way for variant selection may limit the potential of the regularization-based methods EN and BR, which are known for their strength in feature selection. Our follow-up analysis demonstrated including a larger set of genome-wide variants in EN further improved PGS for almost every blood cell trait. These results suggest the EN/BR method as the top choice for PGS development of these (or similar) traits when sufficient individual-level data are available. However, the increased number of input variants can cause increased requirements for the amount of training data, computational resources when using these methods, which could constrain their utilization in PGS development. To address these issues, we also demonstrate that tightening p value thresholds offers a way to not only reduce the size of input variants but also maintain its performance. However, we also find that overly stringent or lenient p value thresholds could cause either deteriorated performance or overfitting problems, so that these should be applied with caution. Our analyses suggest that selecting an appropriate p value threshold through an external validation step could be the key to the better application of these methods in PGS construction.

LDpred2 is specifically designed to consider LD correlation in polygenic prediction, but it still relies on cutoff thresholds to remove the impact of other factors such as low-MAF variants.[29] They can be potential causes for its underperformance or failures in PGS construction, which were demonstrated by the results of LDpred2 on the expanded variant sets. In spite of that, our results did suggest that LDpred2 is still a competitive option for PGS construction (compared with P + T) when appropriate pre-variant selection steps were taken (e.g., applying stringent low-MAF variant filtering and p value thresholding). It is particularly the case when there are only summary-level data available and/or there is a lack of sufficient individual-level data.

Deep learning models MLP and CNN are based on looser model assumptions than the other methods and are capable of modeling more complex relationships among data. The increased model

Table 1. Summary statistics of PGS-sex interaction tests for blood cell traits on INTERVAL

		Effect size			P		
Trait abbreviation	Trait name	Sex (male)	PGS (per SD)	Interaction	Sex	PGS	Interaction
EO%	eosinophil percentage of white cells	0.41	1.30	0.32	<2.2E−16	<2.2E−16	9.60E−11
EO#	eosinophil count	0.013	0.091	0.012	<2.2E−16	<2.2E−16	2.20E−4
HCT	hematocrit	3.68	1.70	0.51	<2.2E−16	<2.2E−16	3.50E−9
HGB	hemoglobin concentration	1.48	0.56	0.24	<2.2E−16	<2.2E−16	<2.2E−16
HLSR#	high light scatter reticulocyte count	0.00061	0.0019	0.00029	<2.2E−16	<2.2E−16	2.03E−5
MCHC	mean corpuscular hemaglobin concentration	0.70	0.74	0.14	<2.2E−16	<2.2E−16	2.63E−5
MONO%	monocyte percentage of white blood cells	0.90	1.73	0.19	<2.2E−16	<2.2E−16	1.37E−5
PCT	plateletcrit	−0.033	0.051	−0.0057	<2.2E−16	<2.2E−16	4.59E−7
PLT#	platelet count	−29.10	56.20	−7.53	<2.2E−16	<2.2E−16	1.71E−12
RET%	reticulocyte fraction of red blood cells	−0.0010	0.30	−0.024	7.34E−1	<2.2E−16	8.84E−4

Interactions between PGS and sex were tested for all of the traits on the INTERVAL cohort by using the multivariate linear regression: $y = \beta_0 + \beta_1 \cdot PGS + \beta_2 \cdot Sex + \beta_3 * PGS * Sex$, where y is the actual trait levels adjusted for technical artifacts, season, age, and the first 10 genetic principal components; PGSs were constructed using EN (p value threshold = 1) on UKB samples and standardized in the model. There are 10 traits whose p values of interaction term passed the Bonferroni significance threshold 10^{-3}, which are listed in the table. SD, standard deviation.

complexity of MLP and CNN did not result in improvements for PGS construction of blood cell traits, indicating that explicit incorporation of non-linearity factors in the two models does not offer an advantage in this setting. However, it is well known that the design of customized network structures plays a pivotal role in addressing a specific task in deep learning, which encourages us to further design and optimize networks beyond these standard structures for PGS construction. Meanwhile, it was noted that the scalability problem of existing deep learning frameworks in ultrahigh-dimensional genotype data will be a major challenge for the widespread application of neural network-based methods in PGS development. Thus, deep learning frameworks based on efficient genotype data format (e.g., bed format in Plink[30]), may represent the future efforts of the area.

We demonstrated that population-based samples can be stratified by the PGSs of these blood cell traits, even for traits exhibiting substantial differences between ages and sexes. These observations may offer therapeutic insights. For example, it is known that some drugs, such as clozapine and dapsone,[31] have neutropenia side effects. The difference between top and bottom quintiles of the neutrophil count (NEUT#) PGS was ∼1,000 NEUTs per microliter; therefore, there may be clinical utility in *a priori* knowledge that an individual may have genetically lowered NEUT# so as to guide pharmacotherapy. It is also well known that blood cell traits are associated with the risk of some complex diseases (e.g., between eosinophil counts and asthma[5]), which indicates that PGS may be useful in follow-up studies on disease risk prediction.

LIMITATIONS OF THE STUDY

MLP and CNN are two of the most common and fundamental deep learning models, and previous studies have demonstrated their potential in genetic prediction.[19,32,33] The present study is limited to two specific but common deep learning models; thus, we cannot make conclusive suggestions on the applications of the whole line of deep learning methods for PGS development of blood cell traits. Taking the optimized MLP/CNN structures further and learning from the characteristics of EN/BR methods may represent the future for designing customized deep learning methods in the field. Also, there are theoretically infinite possibilities for MLP or CNN structures, so we had to restrict the searching within a fixed set of configurations when identifying the optimal PGS model of blood cell traits. The selection for these configurations was based on recommendations in deep learning studies[34] as well as previous findings on the application of neural networks in genetic prediction.[19,32] While the adopted configurations have wide coverage of common MLP/CNN structures, it is still possible that there are other MLP/CNN structures that can construct better PGSs but are not included in the study.

In addition, while the present study focused on PGS development of blood cell traits, the method comparison analysis provides a potentially useful reference for PGS development of other cellular and molecular traits. However, these methods may perform differently for other phenotypes, such as complex diseases with very different genetic architectures. In this case, dedicated studies will be needed to identify an appropriate method for PGS construction of the phenotype.

The extensive sharing of the polygenic basis for blood cell traits and several common diseases was consistent with known trait-level associations and raised potentially fruitful avenues for future translational research. For example, both EO# and NEUT# are important risk factors for rheumatoid arthritis (RA), and their respective PGSs reflected these associations. Knowledge of their shared genetics and corresponding PGSs may enable early stratification of individuals at increased risk

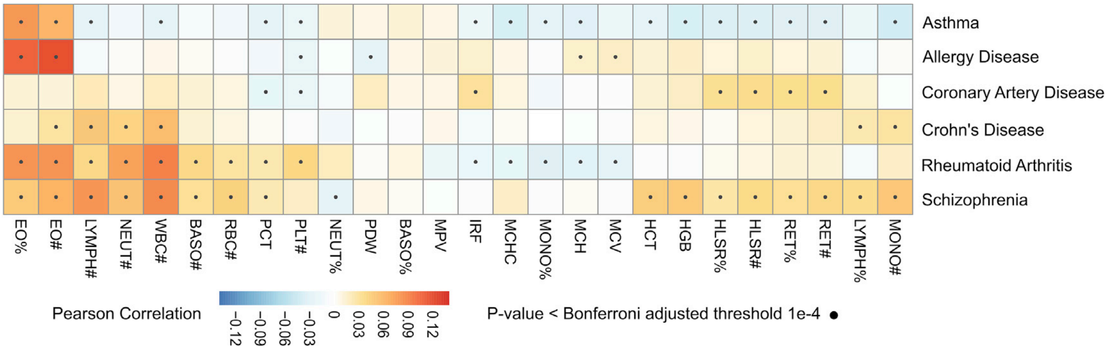

Figure 5. Correlation between PGS for blood cell traits and PGS for 6 common diseases in INTERVAL

PGSs for blood cell traits; diseases were adjusted for the first 10 genetic principal components before the correlation analysis. Pearson *r* correlation analysis was performed between the blood cell trait PGSs and disease PGSs across INTERVAL samples, and the correlation tests with the p value passing the threshold of $p = 10^{-4}$ (Bonferroni adjusted for all trait-disease tests) were deemed significant.

of EO- or NEUT-related RA. Such insights represent new avenues for using PGSs to interrogate disease biology. To facilitate the use of this resource, we have made the blood cell trait PGSs constructed using EN (on the expanded variant set with p value threshold = 1 and trained on UK Biobank) publicly available at the PGS Catalog.[20]

Overall, this study evaluated a variety of learning methods to construct PGSs for blood cell traits using individual-level genotype data. We demonstrate how the learning methods outperform univariate analysis-based methods, including by adjusting the effects of correlation, interaction, and low-MAF variants. This work highlights the importance of moving beyond standard summary statistics-based methods for PGS, particularly as the biobank-scale cohorts are becoming more common. We have made these PGSs available to the community, demonstrated that they can stratify sex- and age-dependent trajectories, and identify their shared polygenic basis with various common diseases. Future studies leveraging the totality of genetic variation (e.g., the full allelic spectrum and difficulty to genotype/sequence loci) for blood cell traits, as identified in recent studies[10] may provide further improvements in the PGSs of these traits and may facilitate further studies evaluating their clinical validity.

STAR★METHODS

Detailed methods are provided in the online version of this paper and include the following:

- KEY RESOURCES TABLE
- RESOURCE AVAILABILITY
  - Lead contact
  - Materials availability
  - Data and code availability
- EXPERIMENTAL MODEL AND SUBJECT DETAILS
  - Study cohorts
- METHOD DETAILS
  - Data quality control
  - Variant selection
  - SNP-SNP correlation and interaction detection
  - Polygenic scoring methods
  - Measurement and hyperparameter tuning
  - Derivation of PGS for disease on INTERVAL
- QUANTIFICATION AND STATISTICAL ANALYSIS

SUPPLEMENTAL INFORMATION

Supplemental information can be found online at https://doi.org/10.1016/j.xgen.2021.100086.

ACKNOWLEDGMENTS

UK Biobank data access was approved under project 13745, and all of the participants gave their informed consent for health research. Participants in the INTERVAL randomized controlled trial were recruited with the active collaboration of NHS Blood and Transplant England (www.nhsbt.nhs.uk), which has supported field work and other elements of the trial. DNA extraction and genotyping were co-funded by the National Institute for Health Research (NIHR), the NIHR BioResource (http://bioresource.nihr.ac.uk), and the NIHR Cambridge Biomedical Research Centre (BRC-1215-20014). The academic coordinating center for INTERVAL was supported by core funding from the NIHR Blood and Transplant Research Unit in Donor Health and Genomics (NIHR BTRU-2014-10024), the UK Medical Research Council (MR/L003120/1), the British Heart Foundation (SP/09/002, RG/13/13/30194, RG/18/13/33946), and the NIHR Cambridge BRC (BRC-1215-20014). (A complete list of the investigators and contributors to the INTERVAL trial is provided in Di Angelantonio, E., Thompson, S.G., Kaptoge, S.K., Moore, C., Walker, M., Armitage, J., Ouwehand, W.H., Roberts, D.J., and Danesh, J.; INTERVAL Trial Group [2017]. Efficiency and safety of varying the frequency of whole blood donation (INTERVAL): a randomised trial of 45 000 donors. Lancet 390, 2360–2371.) The academic coordinating center would like to thank blood donor center staff and blood donors for participating in the INTERVAL trial. This work was supported by Health Data Research UK, which is funded by the UK Medical Research Council, the Engineering and Physical Sciences Research Council, the Economic and Social Research Council, the Department of Health and Social Care (England), the Chief Scientist Office of the Scottish Government Health and Social Care Directorates, the Health and Social Care Research and Development Division of the Welsh government, Public Health Agency (Northern Ireland), and the British Heart Foundation and Wellcome. Y.X. was supported by the UK Economic and Social Research Council (ES/T013192/1). D.V. was funded by the NIHR Blood and Transplant Research Unit in Donor Health and Genomics (NIHR BTRU-2014-10024). S.C.R. is funded by a BHF Programme Grant (RG/18/13/33946). P.A. was funded by the NIHR Blood and Transplant Research Unit in Donor Health and Genomics (NIHR BTRU-2014-10024). T.J. is funded by the NIHR Cambridge Biomedical Research Centre

(BRC-1215-20014). J.G. was supported by a La Trobe University Postgraduate Research Scholarship jointly funded by the Baker Heart and Diabetes Institute and a La Trobe University Full-Fee Research Scholarship. D.J.R. was supported by NHS Blood and Transplant Research and Development funding and the Oxford Biomedical Research Centre (Haematology Theme). J.D. holds a British Heart Foundation Professorship and a NIHR Senior Investigator Award. M.I. is supported by the Munz Chair of Cardiovascular Prediction and Prevention, the Horizon 2020 Research and Innovation Programme 'INTERVENE' (101016775), the UK Economic and Social Research Council (ES/T013192/1) and the NIHR Cambridge Biomedical Research Centre (BRC-1215-20014). This study was supported by the Victorian Government's Operational Infrastructure Support (OIS) program. The views expressed are those of the author(s) and not necessarily those of the NHS, the NIHR, or the Department of Health and Social Care.

AUTHOR CONTRIBUTIONS

M.I. and Y.X. conceived and designed the study. Y.X., D.V., S.C.R., P.A., T.J., and J.G. performed the analyses. A.S.B., W.H.O., D.J.R., E.D.A., J.D., and N.S. provided the data. M.I., N.S., J.D., and A.S.B. supervised the work. Y.X. and M.I. wrote the paper, with input from all of the authors.

DECLARATION OF INTERESTS

P.A. is a full-time employee of Regeneron Pharmaceuticals. A.S.B. has received grants (outside of this work) from AstraZeneca, Bayer, Biogen, BioMarin, Bioverativ, Merck, Novartis, Regeneron, and Sanofi. J.D. reports grants, personal fees, and non-financial support from Merck Sharp & Dohme (MSD); grants, personal fees, and non-financial support from Novartis; grants from Pfizer; and grants from AstraZeneca outside the submitted work. J.D. sits on the International Cardiovascular and Metabolic Advisory Board for Novartis (since 2010), serves on the Steering Committee of UK Biobank (since 2011), is an MRC International Advisory Group (ING) member, London (since 2013), an MRC High Throughput Science 'Omics Panel Member, London (since 2013), a Scientific Advisory Committee member for Sanofi (since 2013), an International Cardiovascular and Metabolism Research and Development Portfolio Committee member for Novartis, and was a member of the Astra Zeneca Genomics Advisory Board (2018).

Received: October 20, 2020
Revised: August 24, 2021
Accepted: December 13, 2021
Published: January 12, 2022

REFERENCES

1. Jensen, F.B. (2009). The dual roles of red blood cells in tissue oxygen delivery: oxygen carriers and regulators of local blood flow. J. Exp. Biol. *212*, 3387–3393.
2. Jenne, C.N., Urrutia, R., and Kubes, P. (2013). Platelets: bridging hemostasis, inflammation, and immunity. Int. J. Lab. Hematol. *35*, 254–261.
3. Nagata, S. (2018). Apoptosis and Clearance of Apoptotic Cells. Annu. Rev. Immunol. *36*, 489–517.
4. Astle, W.J., Elding, H., Jiang, T., Allen, D., Ruklisa, D., Mann, A.L., Mead, D., Bouman, H., Riveros-Mckay, F., Kostadima, M.A., et al. (2016). The Allelic Landscape of Human Blood Cell Trait Variation and Links to Common Complex Disease. Cell *167*, 1415–1429.e19.
5. Castro, M., Zangrilli, J., Wechsler, M.E., Bateman, E.D., Brusselle, G.G., Bardin, P., Murphy, K., Maspero, J.F., O'Brien, C., and Korn, S. (2015). Reslizumab for inadequately controlled asthma with elevated blood eosinophil counts: results from two multicentre, parallel, double-blind, randomised, placebo-controlled, phase 3 trials. Lancet Respir. Med. *3*, 355–366.
6. Sudlow, C., Gallacher, J., Allen, N., Beral, V., Burton, P., Danesh, J., Downey, P., Elliott, P., Green, J., Landray, M., et al. (2015). UK biobank: an open access resource for identifying the causes of a wide range of complex diseases of middle and old age. PLoS Med. *12*, e1001779.
7. Bycroft, C., Freeman, C., Petkova, D., Band, G., Elliott, L.T., Sharp, K., Motyer, A., Vukcevic, D., Delaneau, O., O'Connell, J., et al. (2018). The UK Biobank resource with deep phenotyping and genomic data. Nature *562*, 203–209.
8. Moore, C., Sambrook, J., Walker, M., Tolkien, Z., Kaptoge, S., Allen, D., Mehenny, S., Mant, J., Di Angelantonio, E., Thompson, S.G., et al. (2014). The INTERVAL trial to determine whether intervals between blood donations can be safely and acceptably decreased to optimise blood supply: study protocol for a randomised controlled trial. Trials *15*, 363.
9. Torkamani, A., Wineinger, N.E., and Topol, E.J. (2018). The personal and clinical utility of polygenic risk scores. Nat. Rev. Genet. *19*, 581–590.
10. Vuckovic, D., Bao, E.L., Akbari, P., Lareau, C.A., Mousas, A., Jiang, T., Chen, M.-H., Raffield, L.M., Tardaguila, M., Huffman, J.E., et al.; VA Million Veteran Program (2020). The Polygenic and Monogenic Basis of Blood Traits and Diseases. Cell *182*, 1214–1231.e11.
11. Kim-Hellmuth, S., and Lappalainen, T. (2016). Concerted Genetic Function in Blood Traits. Cell *167*, 1167–1169.
12. Ritchie, S.C., Lambert, S.A., Arnold, M., Teo, S.M., Lim, S., Scepanovic, P., Marten, J., Zahid, S., Chaffin, M., Liu, Y., et al. (2021). Integrative analysis of the plasma proteome and polygenic risk of cardiometabolic diseases. Nat. Metab. *3*, 1476–1483.
13. Chatterjee, N., Shi, J., and García-Closas, M. (2016). Developing and evaluating polygenic risk prediction models for stratified disease prevention. Nat. Rev. Genet. *17*, 392–406.
14. Lambert, S.A., Abraham, G., and Inouye, M. (2019). Towards clinical utility of polygenic risk scores. Hum. Mol. Genet. *28* (*R2*), R133–R142.
15. Wei, Z., Wang, K., Qu, H.Q., Zhang, H., Bradfield, J., Kim, C., Frackleton, E., Hou, C., Glessner, J.T., Chiavacci, R., et al. (2009). From disease association to risk assessment: an optimistic view from genome-wide association studies on type 1 diabetes. PLoS Genet. *5*, e1000678.
16. Abraham, G., Kowalczyk, A., Zobel, J., and Inouye, M. (2013). Performance and robustness of penalized and unpenalized methods for genetic prediction of complex human disease. Genet. Epidemiol. *37*, 184–195.
17. Okser, S., Pahikkala, T., Airola, A., Salakoski, T., Ripatti, S., and Aittokallio, T. (2014). Regularized machine learning in the genetic prediction of complex traits. PLoS Genet. *10*, e1004754.
18. Abraham, G., Tye-Din, J.A., Bhalala, O.G., Kowalczyk, A., Zobel, J., and Inouye, M. (2014). Accurate and robust genomic prediction of celiac disease using statistical learning. PLoS Genet. *10*, e1004137.
19. Bellot, P., de Los Campos, G., and Pérez-Enciso, M. (2018). Can Deep Learning Improve Genomic Prediction of Complex Human Traits? Genetics *210*, 809–819.
20. Lambert, S.A., Gil, L., Jupp, S., Ritchie, S.C., Xu, Y., Buniello, A., McMahon, A., Abraham, G., Chapman, M., Parkinson, H., et al. (2021). The Polygenic Score Catalog as an open database for reproducibility and systematic evaluation. Nat. Genet. *53*, 420–425.
21. Sharp, S.A., Rich, S.S., Wood, A.R., Jones, S.E., Beaumont, R.N., Harrison, J.W., Schneider, D.A., Locke, J.M., Tyrrell, J., Weedon, M.N., et al. (2019). Development and Standardization of an Improved Type 1 Diabetes Genetic Risk Score for Use in Newborn Screening and Incident Diagnosis. Diabetes Care *42*, 200–207.
22. Khramtsova, E.A., Davis, L.K., and Stranger, B.E. (2019). The role of sex in the genomics of human complex traits. Nat. Rev. Genet. *20*, 173–190.
23. Chen, Y., Zhang, Y., Zhao, G., Chen, C., Yang, P., Ye, S., and Tan, X. (2016). Difference in Leukocyte Composition between Women before and after Menopausal Age, and Distinct Sexual Dimorphism. PLoS ONE *11*, e0162953.
24. Cai, H.Q., Catts, V.S., Webster, M.J., Galletly, C., Liu, D., O'Donnell, M., Weickert, T.W., and Weickert, C.S. (2020). Increased macrophages and changed brain endothelial cell gene expression in the frontal cortex of

CellPress
OPEN ACCESS

people with schizophrenia displaying inflammation. Mol. Psychiatry *25*, 761–775.

25. Rosenberg, H.F., Phipps, S., and Foster, P.S. (2007). Eosinophil trafficking in allergy and asthma. J. Allergy Clin. Immunol. *119*, 1303–1310, quiz 1311–1312.

26. Iwamoto, F., Matsuoka, K., Motobayashi, M., Takenaka, K., Kuno, T., Tanaka, K., Tsukui, Y., Kobayashi, S., Yoshida, T., Fujii, T., et al. (2018). Prediction of disease activity of Crohn's disease through fecal calprotectin evaluated by balloon-assisted endoscopy. J. Gastroenterol. Hepatol. *33*, 1984–1989.

27. Shimizu, Y., Kawashiri, S.Y., Yamanashi, H., Koyamatsu, J., Fukui, S., Kondo, H., Tamai, M., Nakamichi, S., and Maeda, T. (2019). Reticulocyte levels have an ambivalent association with hypertension and atherosclerosis in the elderly: a cross-sectional study. Clin. Interv. Aging *14*, 849–857.

28. Özdin, S., and Böke, Ö. (2019). Neutrophil/lymphocyte, platelet/lymphocyte and monocyte/lymphocyte ratios in different stages of schizophrenia. Psychiatry Res. *271*, 131–135.

29. Privé, F., Arbel, J., and Vilhjálmsson, B.J. (2020). LDpred2: better, faster, stronger. Bioinformatics *36*, 5424–5431.

30. Chang, C.C., Chow, C.C., Tellier, L.C., Vattikuti, S., Purcell, S.M., and Lee, J.J. (2015). Second-generation PLINK: rising to the challenge of larger and richer datasets. Gigascience *4*, 7.

31. Moore, D.C. (2016). Drug-induced neutropenia: a focus on rituximab-induced late-onset neutropenia. P T *41*, 765–768.

32. Ma, W., Qiu, Z., Song, J., Li, J., Cheng, Q., Zhai, J., and Ma, C. (2018). A deep convolutional neural network approach for predicting phenotypes from genotypes. Planta *248*, 1307–1318.

33. Abdollahi-Arpanahi, R., Gianola, D., and Peñagaricano, F. (2020). Deep learning versus parametric and ensemble methods for genomic prediction of complex phenotypes. Genet. Sel. Evol. *52*, 12.

34. Bengio, Y., Goodfellow, I.J., and Courville, A. (2017). Deep Learning (MIT Press).

35. Inouye, M., Abraham, G., Nelson, C.P., Wood, A.M., Sweeting, M.J., Dudbridge, F., Lai, F.Y., Kaptoge, S., Brozynska, M., Wang, T., et al.; UK Biobank CardioMetabolic Consortium CHD Working Group (2018). Genomic Risk Prediction of Coronary Artery Disease in 480,000 Adults: Implications for Primary Prevention. J. Am. Coll. Cardiol. *72*, 1883–1893.

36. Ruderfer, D.M., Ripke, S., McQuillin, A., Boocock, J., Stahl, E.A., Pavlides, J.M.W., Mullins, N., Charney, A.W., Ori, A.P.S., Loohuis, L.M.O., et al.; Bipolar Disorder and Schizophrenia Working Group of the Psychiatric Genomics Consortium; Bipolar Disorder and Schizophrenia Working Group of the Psychiatric Genomics Consortium (2018). Genomic Dissection of Bipolar Disorder and Schizophrenia, Including 28 Subphenotypes. Cell *173*, 1705–1715.e16.

37. Liu, J.Z., van Sommeren, S., Huang, H., Ng, S.C., Alberts, R., Takahashi, A., Ripke, S., Lee, J.C., Jostins, L., Shah, T., et al.; International Multiple Sclerosis Genetics Consortium; International IBD Genetics Consortium (2015). Association analyses identify 38 susceptibility loci for inflammatory bowel disease and highlight shared genetic risk across populations. Nat. Genet. *47*, 979–986.

38. Okada, Y., Wu, D., Trynka, G., Raj, T., Terao, C., Ikari, K., Kochi, Y., Ohmura, K., Suzuki, A., Yoshida, S., et al.; RACI Consortium; GARNET Consortium (2014). Genetics of rheumatoid arthritis contributes to biology and drug discovery. Nature *506*, 376–381.

39. Ferreira, M.A., Vonk, J.M., Baurecht, H., Marenholz, I., Tian, C., Hoffman, J.D., Helmer, Q., Tillander, A., Ullemar, V., van Dongen, J., et al.; 23andMe Research Team; AAGC Collaborators; BIOS Consortium; LifeLines Cohort Study (2017). Shared genetic origin of asthma, hay fever and eczema elucidates allergic disease biology. Nat. Genet. *49*, 1752–1757.

40. Demenais, F., Margaritte-Jeannin, P., Barnes, K.C., Cookson, W.O.C., Altmüller, J., Ang, W., Barr, R.G., Beaty, T.H., Becker, A.B., Beilby, J., et al.; Australian Asthma Genetics Consortium (AAGC) Collaborators (2018). Multiancestry association study identifies new asthma risk loci that colocalize with immune-cell enhancer marks. Nat. Genet. *50*, 42–53.

41. Pedregos, F., Varoquaux, G., Gramfort, A., Michel, V., Thirion, B., Grisel, O., Blondel, M., Prettenhofer, P., Weiss, R., Dubourg, V., et al. (2011). Scikit-learn: Machine Learning in Python. J. Mach. Learn. Res. *12*, 2825–2830.

42. Qian, J., Tanigawa, Y., Du, W., Aguirre, M., Chang, C., Tibshirani, R., Rivas, M.A., and Hastie, T. (2020). A fast and scalable framework for large-scale and ultrahigh-dimensional sparse regression with application to the UK Biobank. PLoS Genet. *16*, e1009141.

43. Walter, K., Min, J.L., Huang, J., Crooks, L., Memari, Y., McCarthy, S., Perry, J.R.B., Xu, C., Futema, M., Lawson, D., et al.; UK10K Consortium (2015). The UK10K project identifies rare variants in health and disease. Nature *526*, 82–90.

44. Xu, C., Tachmazidou, I., Walter, K., Ciampi, A., Zeggini, E., and Greenwood, C.M.T.; UK10K Consortium (2014). Estimating genome-wide significance for whole-genome sequencing studies. Genet. Epidemiol. *38*, 281–290.

45. Miller, A.J. (1996). The Convergence of Efroymson's Stepwise Regression Algorithm. Am. Stat. *50*, 180–181.

46. Evans, D.M., Visscher, P.M., and Wray, N.R. (2009). Harnessing the information contained within genome-wide association studies to improve individual prediction of complex disease risk. Hum. Mol. Genet. *18*, 3525–3531.

47. Bishop, C.M. (2006). Pattern Recognition and Machine Learning (Springer).

48. Vilhjálmsson, B.J., Yang, J., Finucane, H.K., Gusev, A., Lindström, S., Ripke, S., Genovese, G., Loh, P.R., Bhatia, G., Do, R., et al.; Schizophrenia Working Group of the Psychiatric Genomics Consortium, Discovery, Biology, and Risk of Inherited Variants in Breast Cancer (DRIVE) study (2015). Modeling Linkage Disequilibrium Increases Accuracy of Polygenic Risk Scores. Am. J. Hum. Genet. *97*, 576–592.

49. Bulik-Sullivan, B.K., Loh, P.R., Finucane, H.K., Ripke, S., Yang, J., Patterson, N., Daly, M.J., Price, A.L., and Neale, B.M.; Schizophrenia Working Group of the Psychiatric Genomics Consortium (2015). LD Score regression distinguishes confounding from polygenicity in genome-wide association studies. Nat. Genet. *47*, 291–295.

50. Finucane, H.K., Bulik-Sullivan, B., Gusev, A., Trynka, G., Reshef, Y., Loh, P.R., Anttila, V., Xu, H., Zang, C., Farh, K., et al.; ReproGen Consortium; Schizophrenia Working Group of the Psychiatric Genomics Consortium; RACI Consortium (2015). Partitioning heritability by functional annotation using genome-wide association summary statistics. Nat. Genet. *47*, 1228–1235.

51. Gardner, M.W., and Dorling, S.R. (1998). Artificial neural networks (the multilayer perceptron) - a review of applications in the atmospheric sciences. Atmos. Environ. *32*, 2627–2636.

52. Angermueller, C., Lee, H.J., Reik, W., and Stegle, O. (2017). DeepCpG: accurate prediction of single-cell DNA methylation states using deep learning. Genome Biol. *18*, 67.

53. Abraham, G., Havulinna, A.S., Bhalala, O.G., Byars, S.G., De Livera, A.M., Yetukuri, L., Tikkanen, E., Perola, M., Schunkert, H., Sijbrands, E.J., et al. (2016). Genomic prediction of coronary heart disease. Eur. Heart J. *37*, 3267–3278.

54. Deloukas, P., Kanoni, S., Willenborg, C., Farrall, M., Assimes, T.L., Thompson, J.R., Ingelsson, E., Saleheen, D., Erdmann, J., Goldstein, B.A., et al.; CARDIoGRAMplusC4D Consortium; DIAGRAM Consortium; CARDIOGENICS Consortium; MuTHER Consortium; Wellcome Trust Case Control Consortium (2013). Large-scale association analysis identifies new risk loci for coronary artery disease. Nat. Genet. *45*, 25–33.

55. Nikpay, M., Goel, A., Won, H.H., Hall, L.M., Willenborg, C., Kanoni, S., Saleheen, D., Kyriakou, T., Nelson, C.P., Hopewell, J.C., et al. (2015). A comprehensive 1,000 Genomes-based genome-wide association meta-analysis of coronary artery disease. Nat. Genet. *47*, 1121–1130.

56. Bycroft, C., Freeman, C., Petkova, D., Band, G., Elliott, L.T., Sharp, K., Motyer, A., Vukcevic, D., Delaneau, O., O'Connell, J., et al. (2017). Genome-wide genetic data on ∼500,000 UK Biobank participants. bioRxiv. https://doi.org/10.1101/166298.

57. Loh, P.R., Danecek, P., Palamara, P.F., Fuchsberger, C., Reshef, Y., Finucane, H.K., Schoenherr, S., Forer, L., McCarthy, S., Abecasis, G.R., et al. (2016). Reference-based phasing using the Haplotype Reference Consortium panel. Nat. Genet. *48*, 1443–1448.

CellPress
OPEN ACCESS

STAR★METHODS

KEY RESOURCES TABLE

REAGENT or RESOURCE	SOURCE	IDENTIFIER
**Deposited data**		
UKB summary statistics of blood cell traits	*Vuckovic et al., 2020*[10]	GWAS Catalog: GCST90002379-GCST90002407
CAD meta-GRS	*Inouye et al., 2018*[35]	PGS Catalog: PGS000018
GWAS summary statistics for schizophrenia	*Ruderfer et al., 2018*[36]	https://figshare.com/articles/dataset/cdg2018-bip-scz/14672019
GWAS summary statistics for Crohn's disease	*Liu et al., 2015*[37]	GWAS Catalog: GCST003044
GWAS summary statistics for rheumatoid arthritis	*Okada et al., 2014*[38]	GWAS Catalog: GCST002318
GWAS summary statistics for allergic disease	*Ferreira et al., 2017*[39]	GWAS Catalog: GCST005038
GWAS summary statistics for asthma	*Demenais et al., 2018*[40]	GWAS Catalog: GCST006862
PGS models	This manuscript	PGS Catalog: PGS000088 - PGS000113
**Software and algorithms**		
R 3.6.3	R Core Team	https://www.r-project.org/
Python 3.6.8	Python Software Foundation	https://www.python.org/
scikit-learn 0.21.2	*Pedregosa et al., 2011*[41]	https://scikit-learn.org/
Keras 2.1.6	N/A	https://keras.io/
SNPNET	*Qian et al., 2020*[42]	https://github.com/junyangq/snpnet
LDpred2	*Privé et al., 2021*[29]	https://privefl.github.io/bigsnpr/articles/LDpred2.html
PLINK 2.0	PLINK Working Group	https://www.cog-genomics.org/plink/2.0/
PLINK 1.9	PLINK Working Group	https://www.cog-genomics.org/plink/1.9/
Bcftools 1.9	N/A	http://samtools.github.io/bcftools/bcftools.html

RESOURCE AVAILABILITY

Lead contact

Further information and requests may be directed to the lead contact Yu Xu (yx322@medschl.cam.ac.uk).

Materials availability

This study did not generate new unique reagents.

Data and code availability

The generated PGS models of blood cell traits have been deposited at the PGS Catalog and are publicly available under accession numbers PGS000088 - PGS000113.

All original code has been deposited at GitHub (https://github.com/xuyu-cam/PGS-BC-Traits-Using-ML-DL).

EXPERIMENTAL MODEL AND SUBJECT DETAILS

Study cohorts

UK Biobank

The UK Biobank is a cohort including 500,000 individuals living in the UK who were recruited between 2006 and 2010, aged between 40 and 69 years at recruitment. The participants with the measurements of the 26 blood cell trait and who were identified as European ancestry based on their genetic component analysis were included in our study. The detailed simple sizes used for training and internal validation of PGS of each blood cell trait after quality control were given in Table S2.

INTERVAL Study

INTERVAL is a randomized trial of 50,000 healthy blood donors, aged 18 years or older at recruitment. The participants with measurements of the 26 considered blood cell trait were included in our study. The detailed simple sizes used for external validation of PGS of each blood cell trait after quality control were given in Table S2.

METHOD DETAILS

Data quality control

This study analyzed 26 different traits across three blood cell types: platelets, red blood cells, and white blood cells (Tables S1 and S2) that were measured in UK Biobank[6,7] and INTERVAL[8] cohorts. As construction and evaluation of PGS are highly dependent on the quality of both phenotype and genotype data used, we adopted the established protocols described in the previous work,[10] adjusting measured values for blood cell trait values to help account for a variety of environmental and technical factors, as well as the first 10 genetic principal components. Technical variables include the time between venepuncture and full blood cell analysis, seasonal effects, center of sample collection, time dependent drift of equipment, systematic differences in equipment; environmental variables include sex, age, and lifestyle factors, including diet, smoking and alcohol consumption. Approaches to quality control and imputation of the genotype data of UK Biobank have been described previously,[7] which filtered the samples to the European-ancestry only; similarly, the quality control and imputation of the genotype data of INTERVAL has been described in the previous work.[4] For algorithmic purposes, any remaining missing genotypes were mean imputed.

Variant selection

To construct PGS for blood cell traits, a key step is to select genetic variants (e.g., SNPs), that are not only significantly associated with the trait but also independently contribute to the trait. Our previous work[10] investigated a range of different variant selection criteria and validated their performance with the P+T method. It was discovered that the conditionally independent variants yield the best predictive power across all the blood cell traits. Thus, this study first adopted the same variant selection strategy for each blood cell trait and used the conditional analysis (CA) variants as inputs to compare the performance of six PGS methods. Below, we describe the steps of the conditional analysis in brief.

A GWAS was first performed for each trait on the UKB cohort to select variants significantly associated with the trait, in which a MAF threshold of 0.005% was applied and an genotype imputation INFO threshold of 0.4. For each variant tested, a genome-wide significance threshold of $p = 8.31 \times 10^{-9}$ was applied as it is widely utilized for common, low frequency and rare variants.[43,44] Details of GWAS for these blood cell traits on UKB have been previously published.[10] Based on these significantly associated variants of each trait, a conditional analysis with a r^2 threshold of 0.9 was further performed to identify the variants that are independently associated with a trait and can best represent the underlying genetic signals of that trait.

The conditional analysis was performed using a stepwise multiple linear regression approach.[4,45] For each blood cell trait, the set of genome wide significant variants was first partitioned into the largest number of blocks such that no pair of blocks are separated by fewer than 5Mb, and no block contains more than 2,500 variants. For each block, variants within the block are tested separately using the multiple-stepwise regression algorithm and independently associated variants are put forward into a larger chromosome wide pool on which a second multiple-stepwise regression algorithm is executed. The multiple-stepwise regression algorithm starts by adding in variants that pass the genome-wide significance threshold ($p = 8.31 \times 10^{-9}$) and have a LD r^2 score lower than 0.9. Then, it fits a multivariate linear regression to remove variants that have a p value larger than the genome-wide significance threshold, which step is iterated until no more variants can be removed from the model. Note that we only keep those CA variants whose genotype data are available on both UKB and INTERVAL studies for the convenience of external tests in this study.

There are PGS methods that are scalable to much higher dimensional genetic data, such as EN and LDpred2, so we further investigated their performance on larger sets of genome-wide variants that were selected with more lenient thresholds. In details, we first selected all the biallelic variants that are shared between UKB and INTERVAL, on which filters, MAF > 0.01, INFO score > 0.4 and variant missing rate < 0.1, were applied to control the quality and total number of selected variants using UKB samples. Then, a LD thinning step was performed on UKB to remove SNP-SNP LD correlations using the *indep-pairwise* method implemented in plink version 2.00[30] at the threshold of $r^2 = 0.5$, which resulted in a total of 1,090,437 variants. Finally, several levels of p value thresholding (i.e., 10^{-6}, 10^{-4}, 10^{-2}, and 1) were applied on these selected variants to form different variant sets for each trait, each of which was incorporated to the CA variant set of the trait for its PGS construction.

SNP-SNP correlation and interaction detection

To investigate capability of learning methods in modeling correlations and interactions, we focused on the CA variant sets and performed correlation and interaction tests between any pairs of CA variants for each trait on the UKB cohort. The coefficient of determination r^2 was used to evaluate the correlation between two variants. The multivariate linear regression: $y = \beta_0 + \beta_1 SNP_1 + \beta_2 SNP_2 + \beta_3 SNP_1 SNP_2$ was employed for interaction tests with the interaction terms passing the threshold of $p = 2.3 \times 10^{-7}$ (Bonferroni adjusted for all tested SNP pairs across the 26 traits) were deemed significant. The term "*interaction*" used here refers to statistical interactions and does not imply (biological) epistasis.

Polygenic scoring methods

We constructed PGS for 26 blood cell traits using a conventional P+T method, summary statistics based learning method LDpred as well as a variety of widely used machine learning and deep learning methods. This subsection describes fundamental aspects of the P+T method, elastic net (EN), Bayesian ridge (BR), LDpred method, multilayer precepton (MLP) and convolutional neural network (CNN) methods.

Pruning and thresholding (P + T)

P+T method assumes that the genetic variants have linear additive effects on PGS of the trait and constructs polygenic scores of a blood cell trait using the weighted sum of genotypes of the selected variants for that trait:[46]

$$\widehat{PGS}_i = \sum_{j \in S} \beta_j \times x_{ij} \qquad \text{(Equation 1)}$$

where S is the set of SNPs that are identified in the variants selection step; β_j *is* the effect size of the SNP j that is obtained through the univariate statistical association tests in the GWAS using the UKB cohort; x_{ij} is the genotype dosage of SNP j of the individual i. As discussed previously, P+T relies on LD pruning and p value thresholding, or maybe other thresholding strategies, for variants selection. The best set of thresholding parameters is usually identified by comparing their performance on a validation set.

Elastic net (EN)

EN also assumes that the variants have linear additive effects on the PGS of a trait, i.e., Equation 1, but the effect sizes of variants are obtained using a different way. These effect sizes are estimated by minimizing the penalized squared loss function:

$$Loss = \sum_{i \in N} \left(y_i - \widehat{PGS}_i\right)^2 + \alpha \times \lambda \times \sum_{j \in S} \left|\beta_j\right| + \frac{\alpha(1-\lambda)}{2} \times \sum_{j \in S} \beta_j^2 \qquad \text{(Equation 2)}$$

in which, N is the set of training samples for a given trait and y_i is the trait level of the training sample i; the second term is L1 norm and the third term is L2 norm; α and λ are coefficients used to control the contribution of L1 and L2 norms in the model, which are usually set via cross-validation. In EN, effect sizes of the variants selected for a trait are jointly estimated which provides an implicit way to model the correlations among these variants, and the use of L1 and L2 norms helps to control model complexity to address the overfitting problem in which L1 controls the sparsity of the model and L2 controls the contribution of each variable. It has been shown that the application of these regularized multivariate models offers an effective way to improve PGS construction in practice.[17,18]

Bayesian ridge (BR)

Similarly, BR also has a linear assumption for the effects of the variants, i.e., Equation 1. Different from EN, BR assumes that PGS of a trait follow a Gaussian distribution, and the prior for effect sizes of variants is also given by a spherical Gaussian:

$$p\left(\widehat{PGS} \middle| \mathbf{x}, \boldsymbol{\beta}, \alpha\right) \sim N\left(\widehat{PGS} \middle| \sum_{j \in S} x_j \beta_j, \alpha^{-1}\right) \qquad \text{(Equation 3)}$$

$$p(\boldsymbol{\beta}|\lambda) \sim N\left(\boldsymbol{\beta}\middle|0, \lambda^{-1}\right) \qquad \text{(Equation 4)}$$

where α and λ are coefficients of the model and subject to two Gamma distribution: Gamma(α_1, α_2) and Gamma(λ_1, λ_2). These two prior Gamma distributions can be set via a validation step. The $\boldsymbol{\beta}$, α, λ are then estimated by maximizing the log of the corresponding posterior distribution with respect to $\boldsymbol{\beta}$ by combining Equations 3 and 4 on the training data.[47]

LDpred

The LDpred method (both version 1 and version 2) also has a fundamental linear assumption as EN and BR. Differently, it considers the prior for effect sizes of variants by a Gaussian mixture model:

$$\beta_j \sim_{iid} \begin{cases} N\left(0, \dfrac{h^2}{Mp}\right) & \textit{with probability } p, \\ 0 & \textit{otherwise} \end{cases} \qquad \text{(Equation 5)}$$

where p is the fraction of causal variants, M the number of variants and h^2 refers to the heritability explained by the genotyped variants. In the first version of LDpred method,[48] the h^2 is estimated by a constrained LD-score regression[49,50] and a list of optional

values for p were recommended for testing on a validation set. With a pair of given h^2 and p, effect sizes of variants are estimated via a Markov chain Monte Carlo (MCMC) method using the summary statistics and a variants-variants correlation matrix (learned from a reference panel). The latest LDpred2[29] extends the LDpred with a couple of new features to offer better software stability, efficiency and model performance. For example, larger window size is introduced to allow for variants correlation modeling in long-range LD regions; a sparse learning option is introduced to allow to fit variant effects to zeros and produce a sparse vector of effects.

Multilayer perceptron (MLP)

MLP is also named Deep Forward Neural Networks. Unlike other statistical learning methods, e.g., EN and BR, MLP makes no prior assumptions on the data distribution and can be trained to approximate virtually any smooth, measurable functions including non-linear functions.[51] A MLP typically consists of many different functions (or neurons) which are composed through a directed acyclic graph.[34] Figure S4 shows an example of a three-layer MLP in which the first layer is known as input layer consisting of the input features, i.e., SNPs in the context of this study; the last layer outputs the final result of the model and the layer(s) in between are called hidden layer(s). A function node in hidden and output layers typically transforms the inputs from the previous layer with a weighted linear sum followed by an activation function.[19] For example, f^1 in Figure S4 can be represented as:

$$f^1(SNP_1, SNP_2, SNP_3) = f^{act}(SNP_1 \times w_{11} + SNP_2 \times w_{12} + SNP_3 \times w_{13} + b_{10}) \quad \text{(Equation 6)}$$

where w_{11}, w_{12} and w_{13}, are weights of the three inputs of function f^1 and b_{10} is the intercept (or bias); SNP_1, SNP_2 and SNP_3 are the genotype dosages of three SNPs in our context; f^{act} is an activation function which typically plays the role of introducing non-linearity into the model. Thus, the network architecture and its components of an MLP, e.g., activation function, determine a linear/non-linear mapping space, from which a model, i.e., all the weights across the given network that can best represent the data, is supposed to be learned. Details on the selection of network architectures for this study are given in the next subsection. This learning process is typically implemented by minimizing the difference, i.e., cost function, between the training data and the model distribution, through a back-propagation algorithm.[34]

Convolutional neural networks (CNNs)

CNNs are a specialized neural network for processing data that have a grid-like topology,[34] e.g., time-series data, image data, genome sequence data.[52] As regularized versions of MLPs, CNNs construct its hidden layers using convolutional and pooling operations which are usually followed by fully connected layers and the output layer. The convolution operation limits the number of input units for an output unit by using kernels, and leads to a sparse connectivity of the network, which allows us to store fewer parameters and largely improve statistical efficiency. A typical convolutional layer in CNNs performs multiple convolutions in parallel which lead to multiple representations of the input units. To help generalize these representations and reduce the chance of overfitting, a pooling layer is usually followed to replace each representation at a certain location with a summary statistic of the nearby output units.[34] There are different pooling operations that can be applied based on different application context, e.g., max pooling and average pooling. Figure S5 shows an example of a simple one-dimensional CNN with illustrations on convolution and pooling operations.

Measurement and hyperparameter tuning

We used Pearson r to measure the performance of various polygenic scoring methods. For each trait and each learning method, we randomly and equally partitioned the UKB samples into 5 portions, from which any 4 portions (80% of the samples) were used as training data to learn a model, and test the respective model's performance on the remaining 20% of UKB samples, as well as an external validation using the whole INTERVAL cohort. For each learning method and each trait, we obtained 5 different models, each with a performance measurement for both the internal UKB test and the external INTERVAL test. By doing so, the training and internal testing covered the whole UKB cohort, affording an effective way to avoid evaluation bias. The P+T method was also tested on the five different UKB testing sets, and the whole INTERVAL cohort.

Hyperparameter turning is a crucial step for machine learning and deep learning methods as the choice of hyperparameters can greatly influence the model performance. In this study, we employed SNPNET[42] to implement EN method, in which α was set to 0.5 and 10% of the training samples were used as a validation set to tune λ for each trait and each variant set. To identify two appropriate gamma distributions in BR i.e., the selection of α_1, α_2, λ_1 and λ_2, a grid search across the set [-10^{10}, -10^5,-10, 0, 10, 10^5, 10^{10}] was conducted on the training set in which 10% of the samples were used as a validation set. BR was implemented using the scikit-learn package.[41] This study applied the commonly used and top performing grid search option in LDpred2 to learn PGS of these blood cell traits. Summary statistics from GWAS in the variants selection step, and the 102 default options of hyper-parameters for h^2, p, and *sparsity* were applied when running LDpred2. Randomly selected 10,000 samples from training data were used to obtain the variants-variants correlation matrix and all the training samples for each trait were used to validate the performance of different hyperparameter combinations for the optimal model selection.

As this work is, to our knowledge, the first attempt to employ MLPs and CNNs for genomic prediction of blood cell traits, there was no prior information that could be used for the design of network architecture for this task. Therefore, similar to the previous work,[19] we used a genetic algorithm to search for the optimal MLP and CNN architectures as well as other hyperparameters, e.g., the number of layers, the number of neurons at each layer, activation functions, optimizers, dropouts, etc., on the training set, in which 10% of the samples were used as a validation set. MLPs and CNNs were implemented using Keras (keras.io).

Derivation of PGS for disease on INTERVAL

The polygenic risk score used for coronary artery disease (CAD) was our previously published CAD meta-GRS;[35] a polygenic score comprising 1.75 million variants derived from a meta-analysis of three PGS for CAD in UK Biobank. Briefly, the three meta-analyzed CAD PGS were: (1) an earlier PGS[53] comprising 46,000 metabochip variants and their log odds for CAD in the 2013 CARDIoGRAMplusC4D consortium GWAS meta-analysis;[54] (2) a PGS comprising 202 variants whose association with CAD in the 2015 CARDIoGRAMplusC4D consortium GWAS meta-analysis[55] were significant at a false discovery rate (FDR) < 0.05; and (3) a genome-wide PGS derived from the same summary statistics[55] LD-thinned at $r^2 = 0.9$ threshold in UK Biobank (version 2 genotype data, imputed to the HRC panel only).

PGS for schizophrenia, Crohn's disease, rheumatoid arthritis, allergic disease and asthma were derived from summary statistics from their respective genome wide association studies (GWAS) by filtering to variants that overlapped with a set of 2.3 million linkage disequilibrium (LD)-thinned ($r^2 < 0.9$), high-confidence (imputation INFO score > 0.4), common (MAF > 1%), unambiguous SNPs (A/T and G/C SNPs excluded) in the UK Biobank version 3 genotype data[6,56] (imputed to the 1000 genomes, UK10K, and haplotype reference consortium (HRC) panels[57]). GWAS summary statistics used for schizophrenia, Crohn's disease, rheumatoid arthritis, allergic disease, asthma were those published in the previous works.[36–40]

Levels of each PGS in each INTERVAL participant were calculated using the *score* method implemented in plink version 2.00.[30] In the case of missing genotypes, the frequency of the effect allele in INTERVAL was used in its place. For each PGS, these total sums were subsequently standardized to have mean of 0 and standard deviation 1 across all INTERVAL participants. Variants with complementary alleles (e.g., A/T and G/C variants) were excluded to avoid incorrect effect allele matching due to strand ambiguity. Where there were duplicate variants the one with the highest INFO score was kept. In total, 54,069,889 variants passed QC for PGS calculation of these diseases.

QUANTIFICATION AND STATISTICAL ANALYSIS

The quantitative and statistical analyses are described in the relevant sections of the Method details or in the table and figure legends.

Cell Genomics, Volume *2*

Supplemental information

Machine learning optimized polygenic scores for blood cell traits identify sex-specific trajectories and genetic correlations with disease

Yu Xu, Dragana Vuckovic, Scott C. Ritchie, Parsa Akbari, Tao Jiang, Jason Grealey, Adam S. Butterworth, Willem H. Ouwehand, David J. Roberts, Emanuele Di Angelantonio, John Danesh, Nicole Soranzo, and Michael Inouye

Supplementary Tables and Figures

Table S1. Summary of measurement methods for the 26 blood cell traits, related to STAR Methods

Cell Type	Standard Abbreviation	Long Name	Unit	Description	Coulter LH 700 Series (UK Biobank)		Sysmex XN-1000 (INTERVAL)	
					Measured / Derived	Determination	Measured / Derived	Determination
Platelet	PLT#	Platelet count	per nL	Count of platelets per unit volume of blood	Measured	Impedance	Measured	Flow cytometry gate (impedance for missing data points)
	MPV	Mean platelet volume	fL	Mean volume of platelets	Derived	(PCT/PLT#)×10000	Derived	(PCT/PLT#)×10000
	PDW	Platelet distribution width	fL	The spread of the platelet volume distribution. Note that Sysmex and Coulter use different statistics to measure spread.	Measured	Impedance: Coefficient of variation of platelet volume distribution	Measured	Impedance: width at 20% peak height of platelet volume histogram.
	PCT	Plateletcrit	%	Volume fraction of blood occupied by platelets	Measured	Impedance	Measured	Impedance
Mature red cell	RBC#	Red blood cell count	per pL	Count of red blood cells per unit volume of blood	Measured	Impedance	Measured	Impedance
	MCV	Mean corpuscular volume	fL	Mean volume of red blood cells	Derived	(HCT/RBC#)×10	Derived	(HCT/RBC#)×10
	HCT	Hematocrit	%	Volume fraction of blood occupied by red cells	Measured	Impedance	Measured	Impedance
	MCH	Mean corpuscular hemoglobin	pg	Average mass of hemoglobin per red cell	Derived	(HGB/RBC#)×10	Derived	(HGB/RBC#)×10
	MCHC	Mean corpuscular hemoglobin concentration	g/dL	Concentration of hemoglobin with respect to unit of volume occupied by red cells	Derived	(HGB/HCT)×100	Derived	(HGB/HCT)×100
	HGB	Hemoglobin concentration	g/dL	Concentration of hemoglobin with respect to unit of volume of blood	Measured	Light absorbance	Measured	Light absorbance
Immature red cell	RET#	Reticulocyte count	pL	Count of reticulocytes per unit volume of blood	Derived	(RET%×RBC#)/100	Derived	(RET%×RBC#)/100
	RET%	Reticulocyte fraction of red cells	%	Percentage of red blood cells that are reticulocytes	Measured	Flow cytometry/impedance	Measured	Flow cytometry gates
	IRF	Immature fraction of reticulocytes	-	Fraction of reticulocytes with high RNA content, as measured by light scatter	Derived	HLSR#/RET#	Measured	Flow cytometry gates

Cell Type	Standard Abbreviation	Long Name	Unit	Description	Coulter LH 700 Series (UK Biobank)		Sysmex XN-1000 (INTERVAL)	
					Measured / Derived	Determination	Measured / Derived	Determination
Immature red cell	HLSR#	High light scatter reticulocyte count	per pL	Count of high RNA content (immature) reticulocytes per unit volume of blood	Derived	(HLSR%×RBC#)/100%	Derived	IRF×RET#
	HLSR%	High light scatter reticulocyte percentage of red cells	%	Immature reticulocyte count as a percentage of red blood cell count	Measured	Flow cytometry/impedance gates	Derived	(HLSR#/RBC#)×100%
Myeloid white cell	MONO#	Monocyte count	per nL	Count of monocytes per unit volume of blood	Derived	(MONO%×WBC#)/100%	Derived	(MONO%×WBC#)/100%
	NEUT#	Neutrophil count	per nL	Count of neutrophils per unit volume of blood	Derived	(NEUT%×WBC#)/100%	Derived	(NEUT%×WBC#)/100%
	EO#	Eosinophil count	per nL	Count of eosinophils per unit volume of blood	Derived	(EO%×WBC#)/100%	Derived	(EO%×WBC#)/100%
	BASO#	Basophil count	per nL	Count of basophils per unit volume of blood	Derived	(BASO%×WBC#)/100%	Derived	(BASO%×WBC#)/100%
Lymphoid white cell	LYMPH#	Lymphocyte count	per nL	Aggregate count of lymphoid cells per unit volume of blood	Derived	(LYMPH%×WBC#)/100%	Derived	(LYMPH%×WBC#)/100%
Compound white cell	WBC#	White blood cell count	per nL	Aggregate count of white cells per unit volume of blood	Measured	Impedance	Measured	Flow cytometry gates
	MONO%	Monocyte percentage of white cells	%	Percentage of white cells that are monocytes	Measured	Flow cytometry gates	Measured	Flow cytometry gates
	NEUT%	Neutrophil percentage of white cells	%	Percentage of white cells that are neutrophils	Measured	Flow cytometry gates	Measured	Flow cytometry gates
	EO%	Eosinophil percentage of white cells	%	Percentage of white cells that are eosinophils	Measured	Flow cytometry gates	Measured	Flow cytometry gates
	BASO%	Basophil percentage of white cells	%	Percentage of white cells that are basophils	Measured	Flow cytometry gates	Measured	Flow cytometry gates
	LYMPH%	Lymphocyte percentage of white cells	%	Percentage of white cells that are lymphocytes	Measured	Flow cytometry gates	Measured	Flow cytometry gates

Table S2. The number of samples and conditional analysis variants used in UK biobank and INTERVAL for each blood cell trait, related to STAR Methods. This table presents the number of valid samples after quality control and the number of variants selected in conditional analysis for each trait.

Trait	Number of Valid Samples		Number of Variants
	UK Biobank	INTERVAL	
PLT#	391232	38939	762
MPV	391598	37224	681
PDW	391450	37262	579
PCT	390803	37306	726
RBC#	408069	40262	707
MCV	407157	40080	739
HCT	408112	40340	513
MCH	406517	40108	682
MCHC	407850	40265	252
HGB	407739	40329	532
RET#	396720	40253	590
RET%	396811	40286	572
IRF	396408	40227	390
HLSR#	400334	40244	605
HLSR%	400438	40225	594
MONO#	403994	39177	674
NEUT#	406788	39138	512
EO#	406470	40276	623
BASO#	404718	39986	198
LYMPH#	407277	39191	639
WBC#	408032	40466	659
MONO%	403136	39189	583
NEUT%	407114	39190	452
EO%	406417	40326	589
BASO%	404532	40133	160
LYMPH%	407319	39178	489

Figure S1. Comparison of CA variant effect sizes between GWAS and EN/BR method, related to Figure 2. EN and BR generated almost the same effect sizes for conditional analysis variants of all the traits, thus for simplicity, this figure only compares the variant effect sizes between EN and the univariant analysis in GWAS. The mean of the 5 effect sizes in the 5 trained EN models for each variant is used as the variant effect size of EN in this figure. The variants whose MAF is smaller than 1% are marked with triangles and others are marked with circles. Those variants that were detected with interactions are marked in red, and variants that were correlated with others with $r^2 > 0.1$ are marked in yellow. If variants fall in both of the scenarios, they are marked in green. Any other variants are marked in blue.

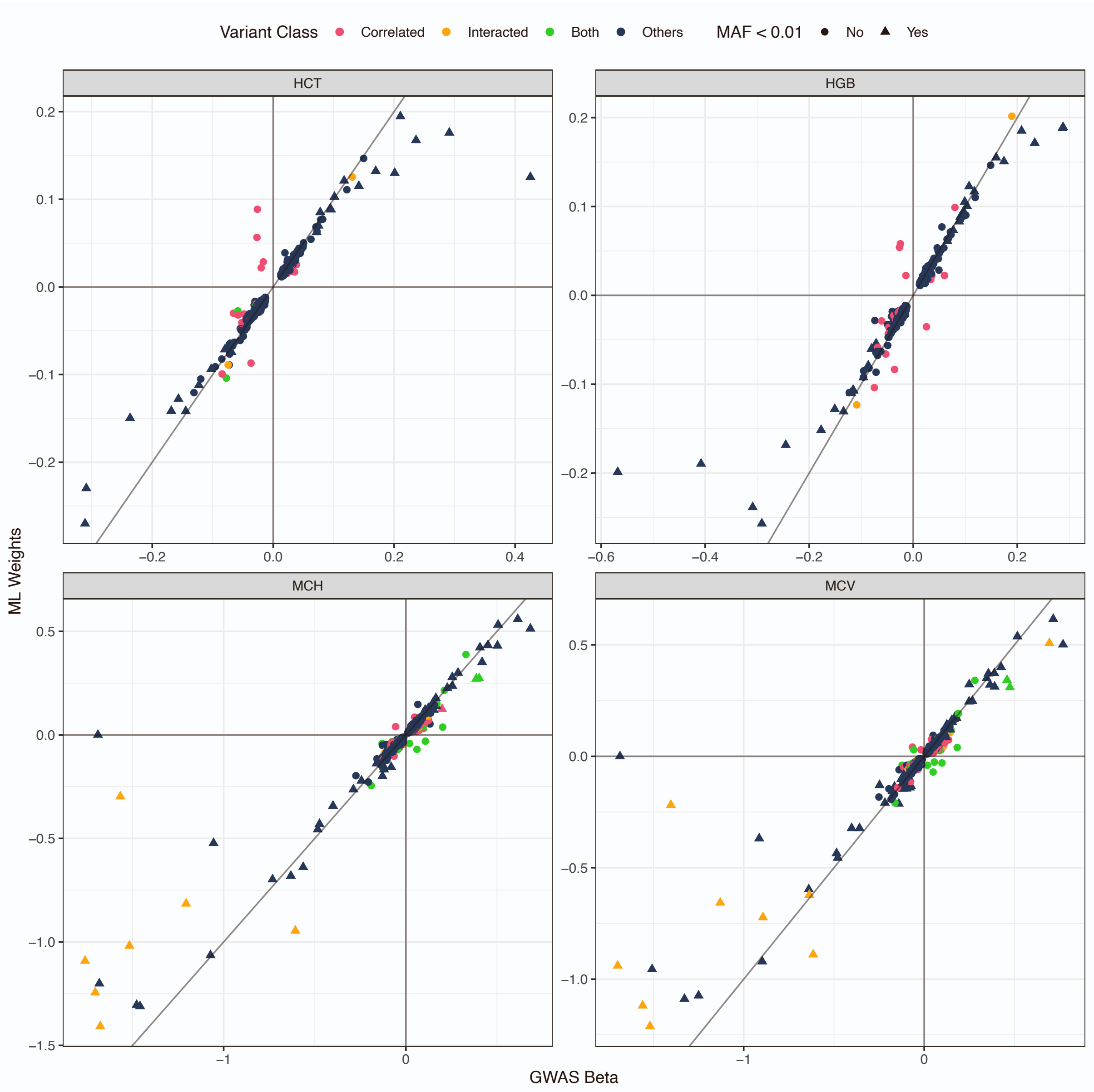

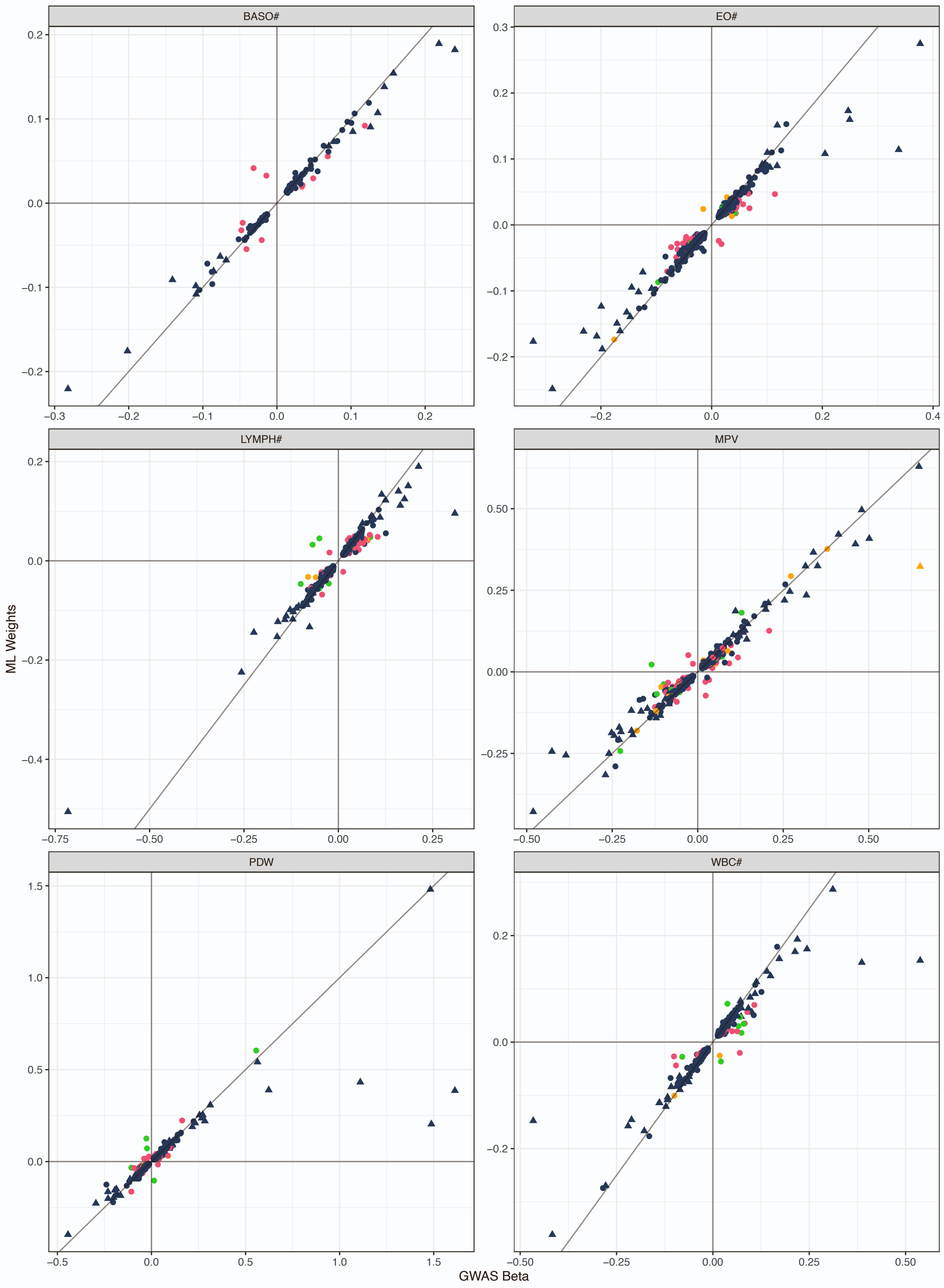

BASO#
EO#
LYMPH#
MPV
PDW
WBC#
ML Weights
GWAS Beta

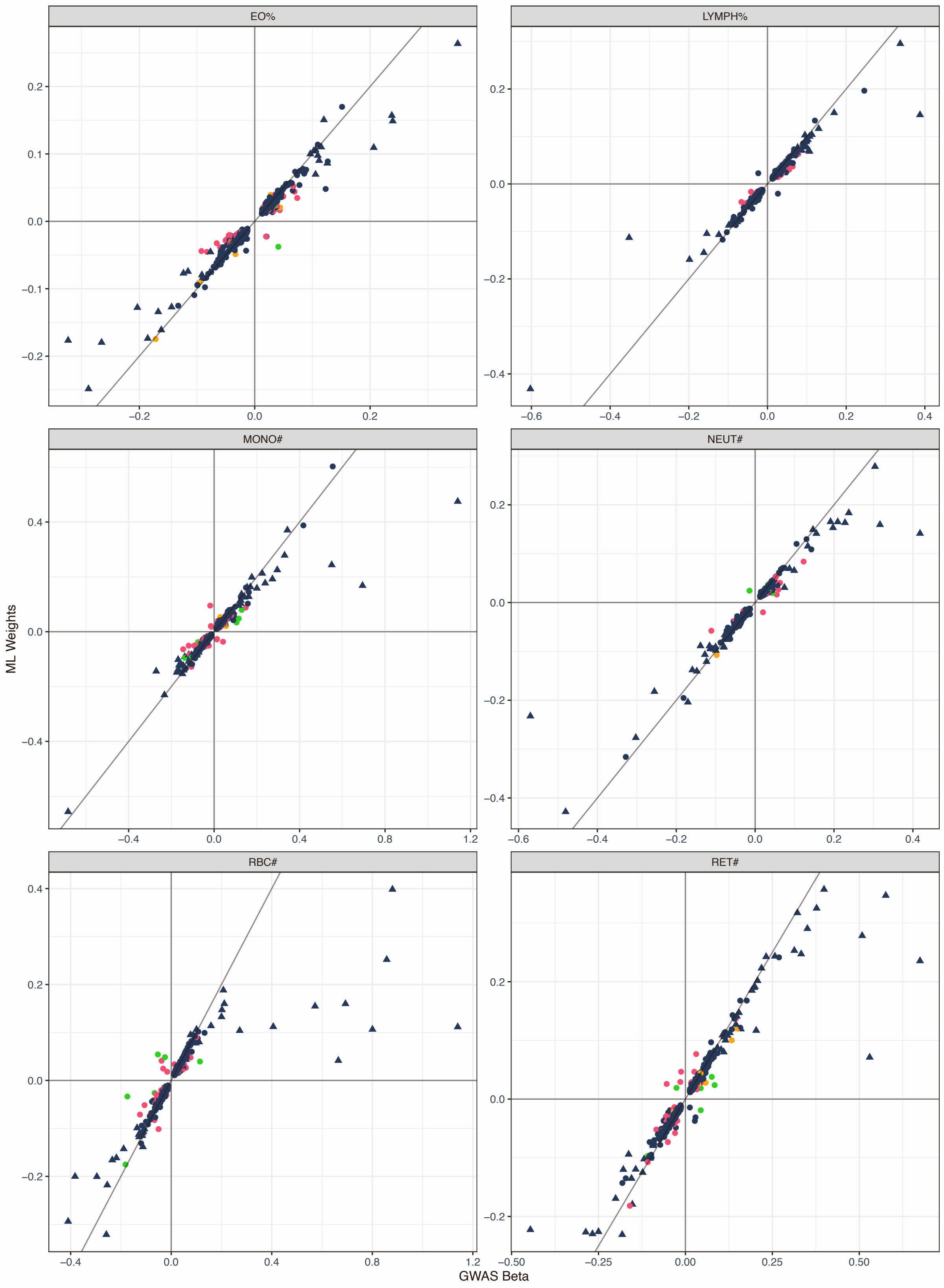

EO%
LYMPH%
MONO#
NEUT#
RBC#
RET#
ML Weights
GWAS Beta

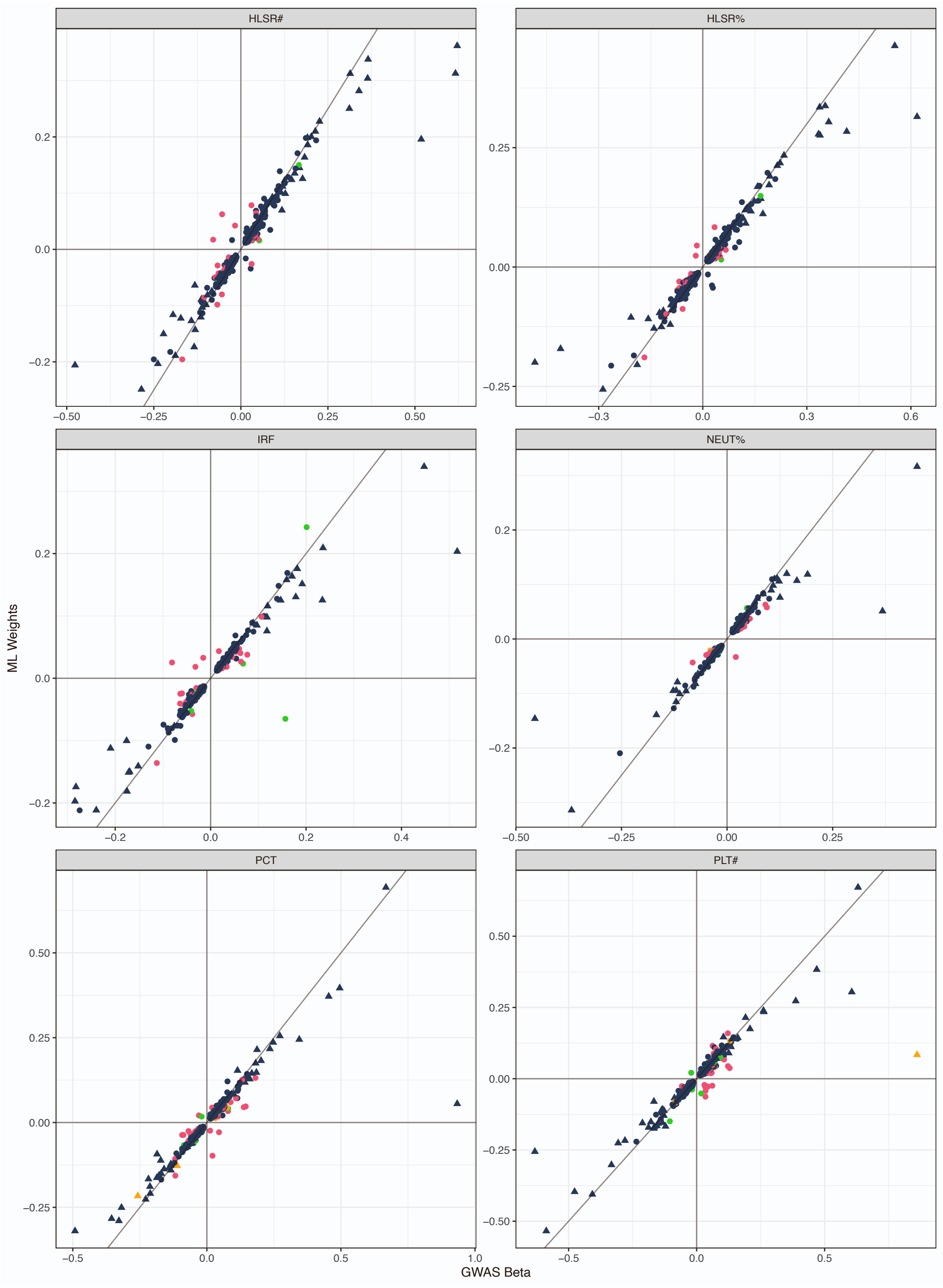

HLSR#
HLSR%
IRF
NEUT%
PCT
PLT#
ML Weights
GWAS Beta

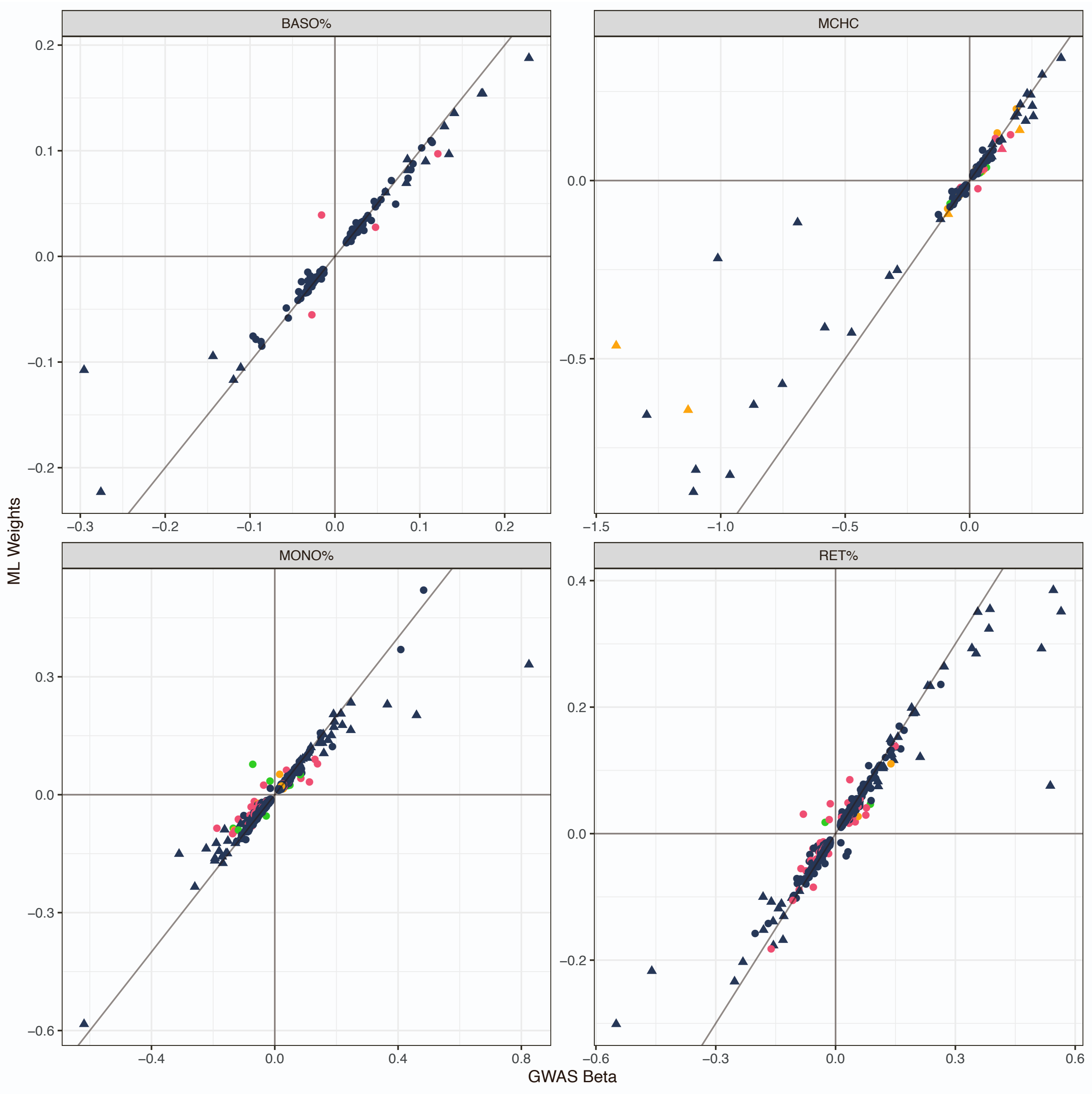

BASO%
MCHC
MONO%
RET%
ML Weights
GWAS Beta

Figure S2. Performance of P+T, EN and LDpred2 methods on different variant sets in UKB, related to Figure 3.

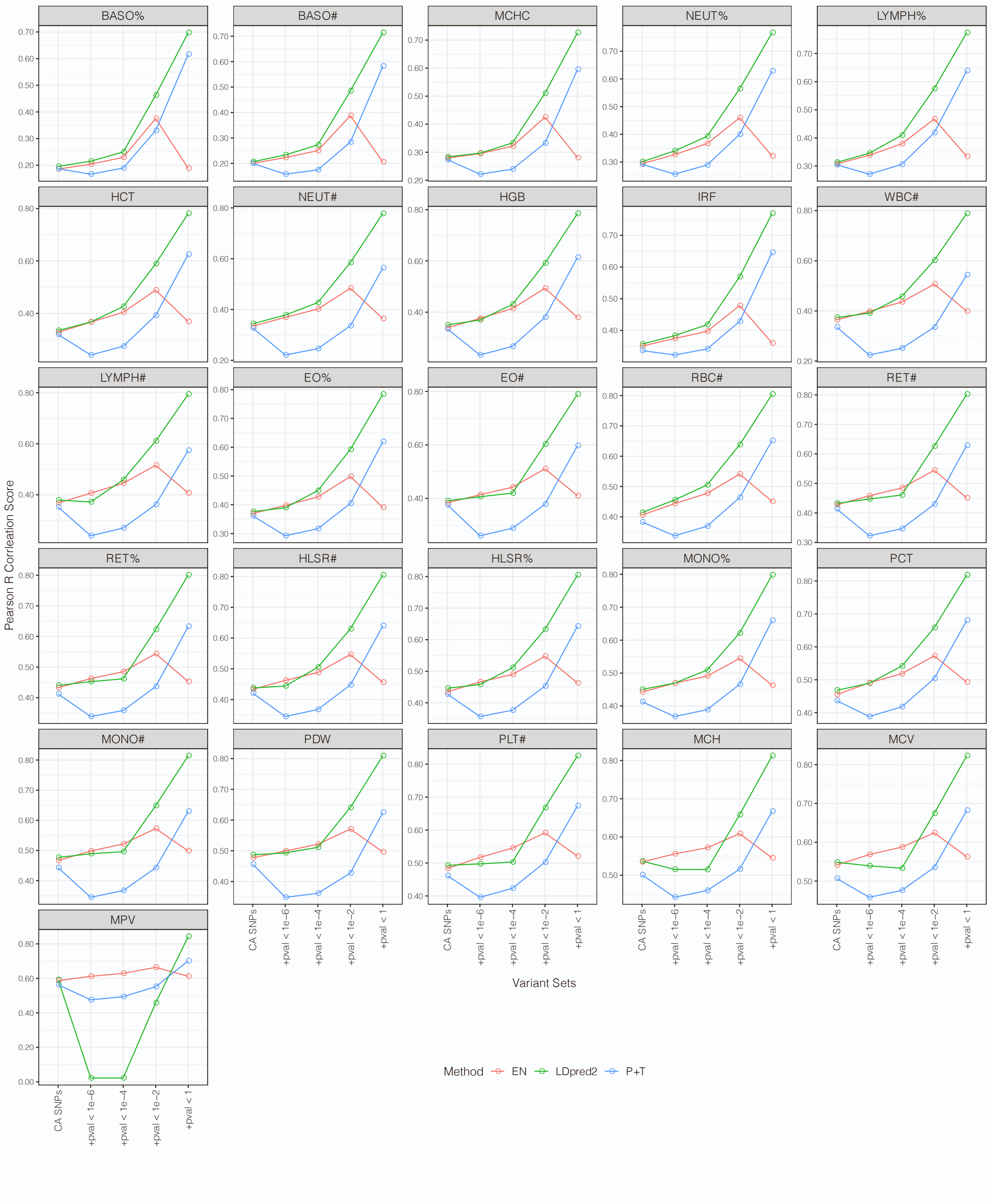

Figure S3. Trait levels by quintiles of EN-trained trait PGSs in men and women for the other 23 blood cell traits on INTERVAL, related to Figure 4. The traits are ordered by their PGS *r* scores (trained using EN on the largest variant set) in INTERVAL.

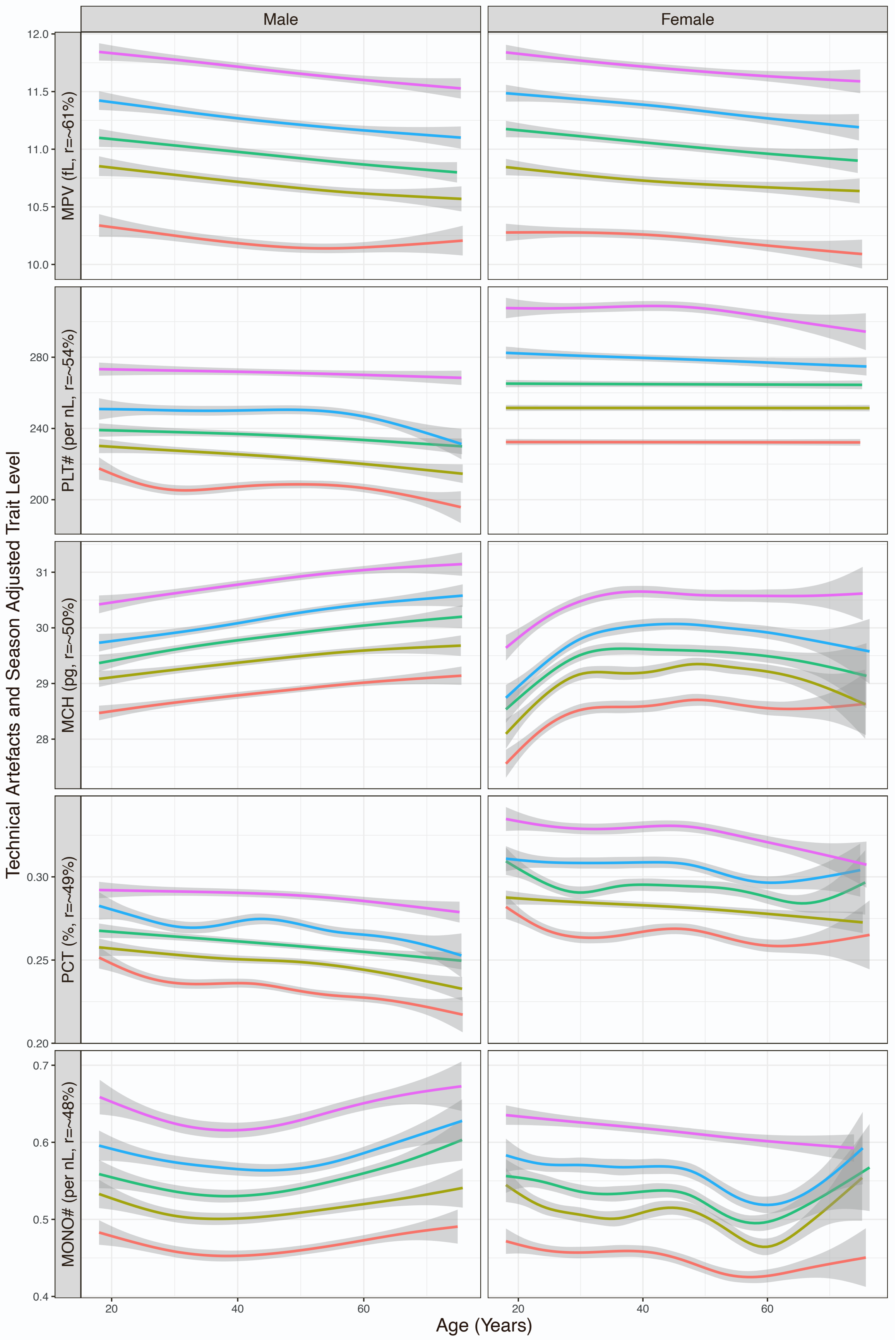

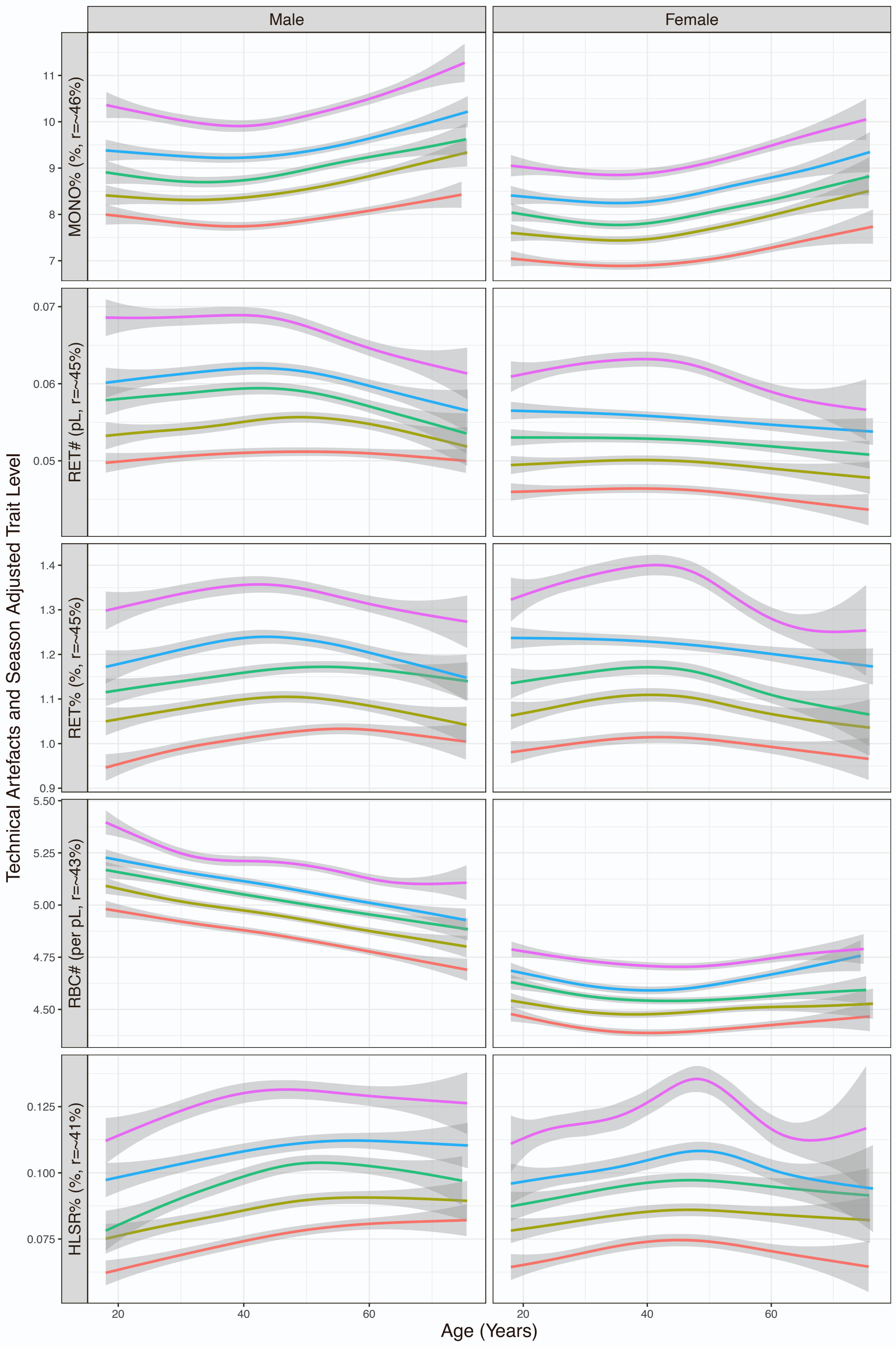
Male
Female
MONO% (%, r=~46%)
RET# (pL, r=~45%)
RET% (%, r=~45%)
RBC# (per pL, r=~43%)
HLSR% (%, r=~41%)
Technical Artefacts and Season Adjusted Trait Level
Age (Years)

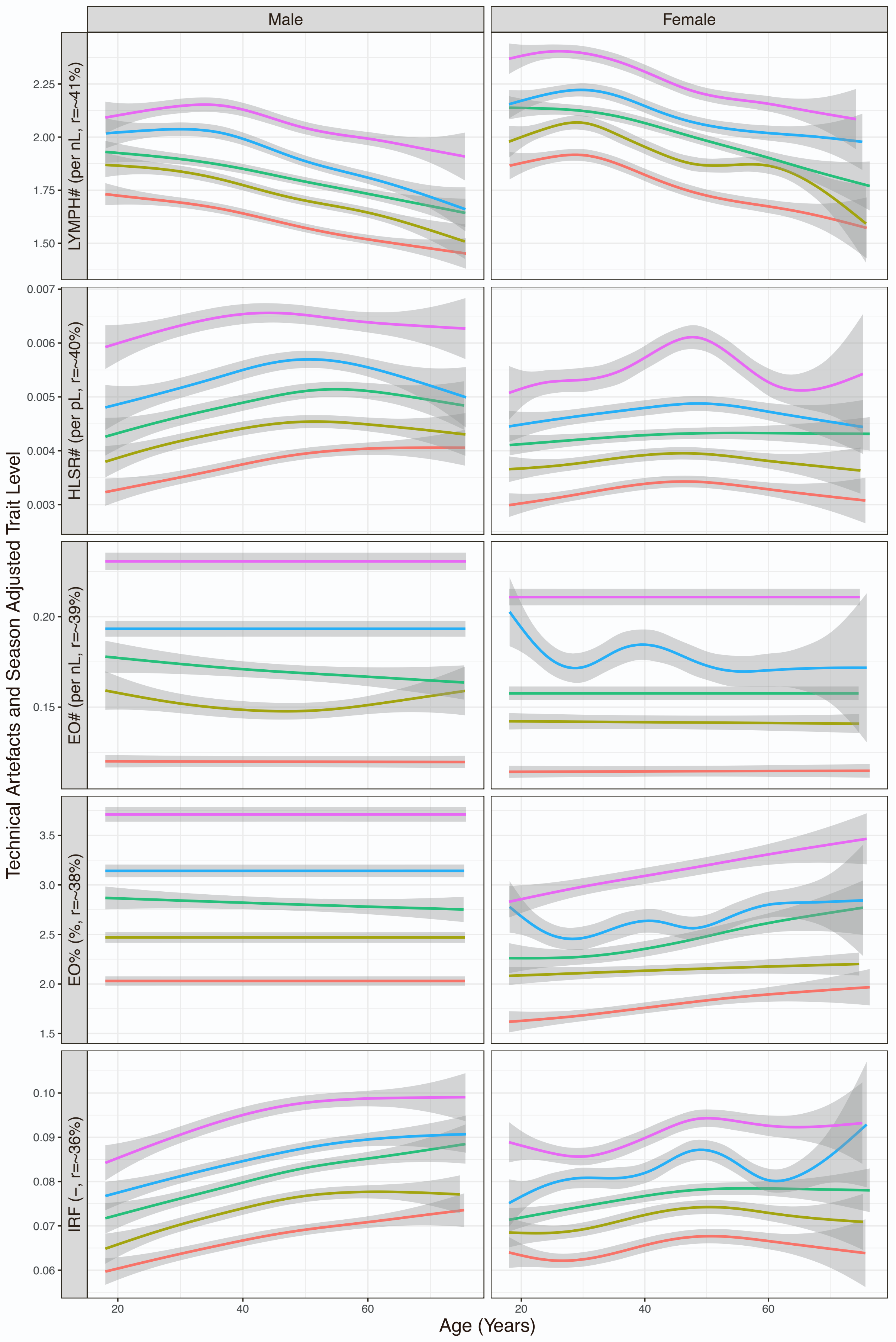

Male
Female
LYMPH# (per nL, r=~41%)
HLSR# (per pL, r=~40%)
EO# (per nL, r=~39%)
EO% (%, r=~38%)
IRF (–, r=~36%)
Technical Artefacts and Season Adjusted Trait Level
Age (Years)
2.25
2.00
1.75
1.50
0.007
0.006
0.005
0.004
0.003
0.20
0.15
3.5
3.0
2.5
2.0
1.5
0.10
0.09
0.08
0.07
0.06
20
40
60

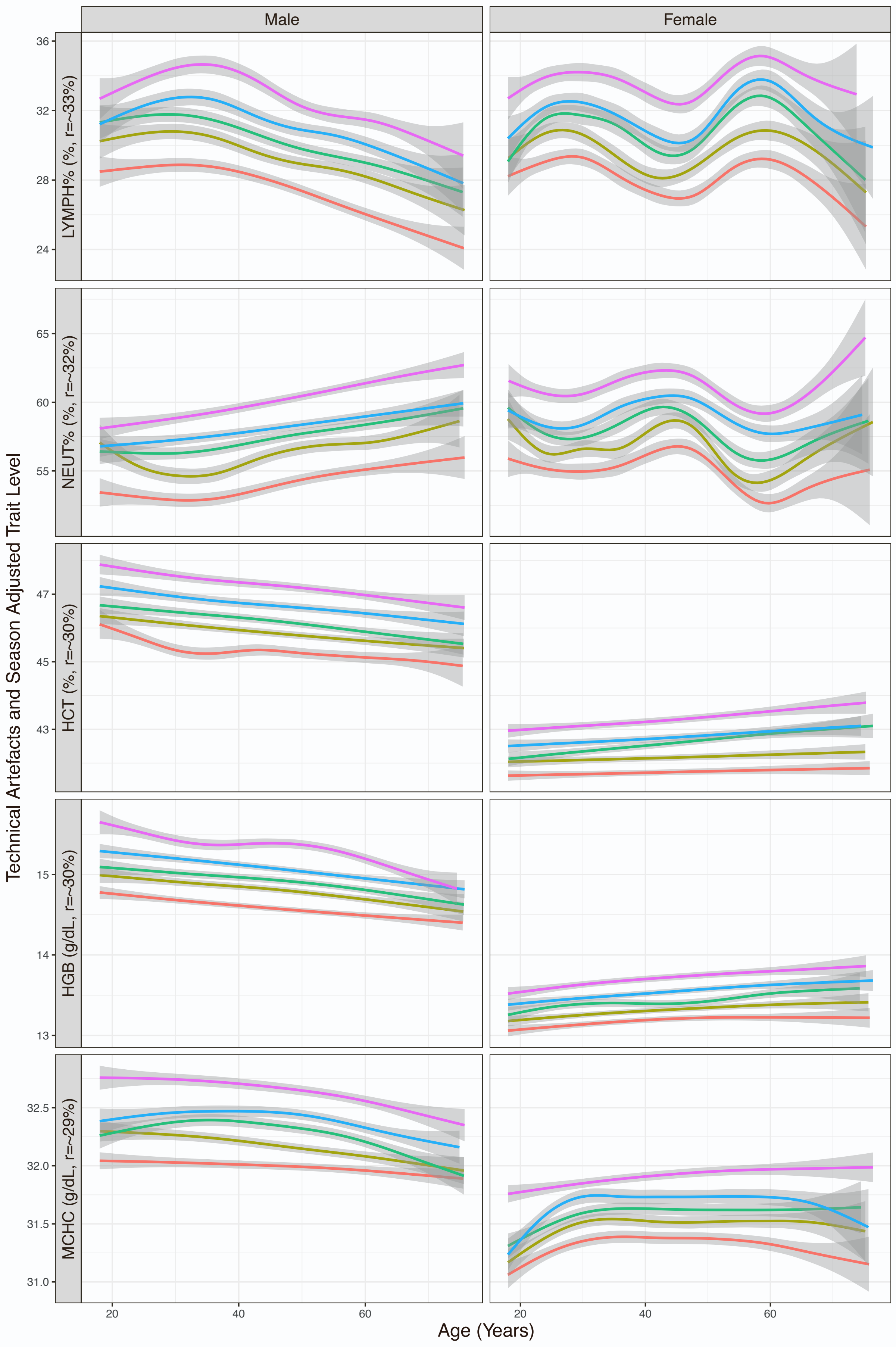

Male
Female
LYMPH% (%, r=~33%)
NEUT% (%, r=~32%)
HCT (%, r=~30%)
HGB (g/dL, r=~30%)
MCHC (g/dL, r=~29%)
Technical Artefacts and Season Adjusted Trait Level
Age (Years)

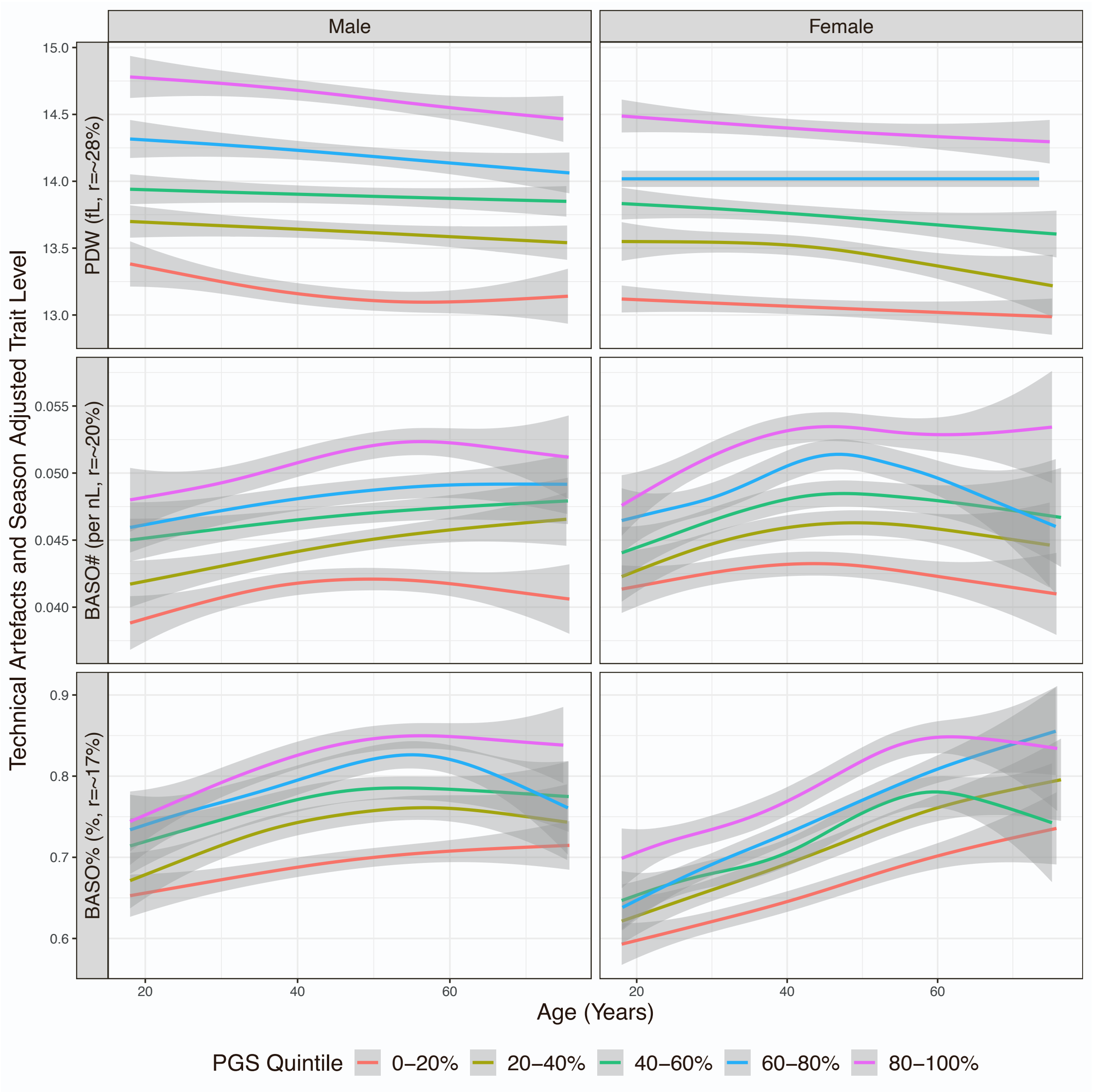

Male
Female
Technical Artefacts and Season Adjusted Trait Level
PDW (fL, r=~28%)
BASO# (per nL, r=~20%)
BASO% (%, r=~17%)
15.0
14.5
14.0
13.5
13.0
0.055
0.050
0.045
0.040
0.9
0.8
0.7
0.6
20
40
60
Age (Years)
PGS Quintile
0–20%
20–40%
40–60%
60–80%
80–100%

Figure S4. An example of a three-layer MLP, related to STAR Methods. The output $y = f^3(f^1(SNP_1, SNP_2, SNP_3), f^2(SNP_1, SNP_2, SNP_3))$

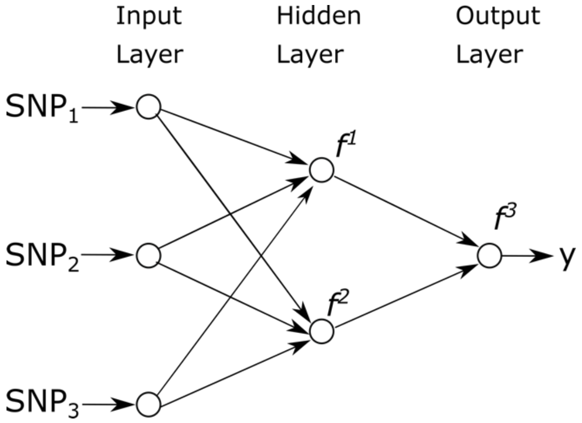

Figure S5. (a) An example of a one-dimensional CNN. (b) An example of a convolution operation. (c) An example of a max pooling operation, related to STAR Methods. The convolution kernel in (b) has a size of 1*2 and operates with a stride of 1. The max pooling filter in (c) has a size 1*2 and operates with a stride of 1. The CNN in (a) has an input of a one-dimensional vector with *n* units, and has a convolution layer and a pooling layer. The dimension *m* of a newly generated representation via a convolution operation relies on the size of the kernel being applied as well as other possible factors, e.g. padding approaches, and the number of new representations *l* is equivalent to the number of kernels used in the model. The dimension *k* of a new representation after pooling is decided by the filter size being used.

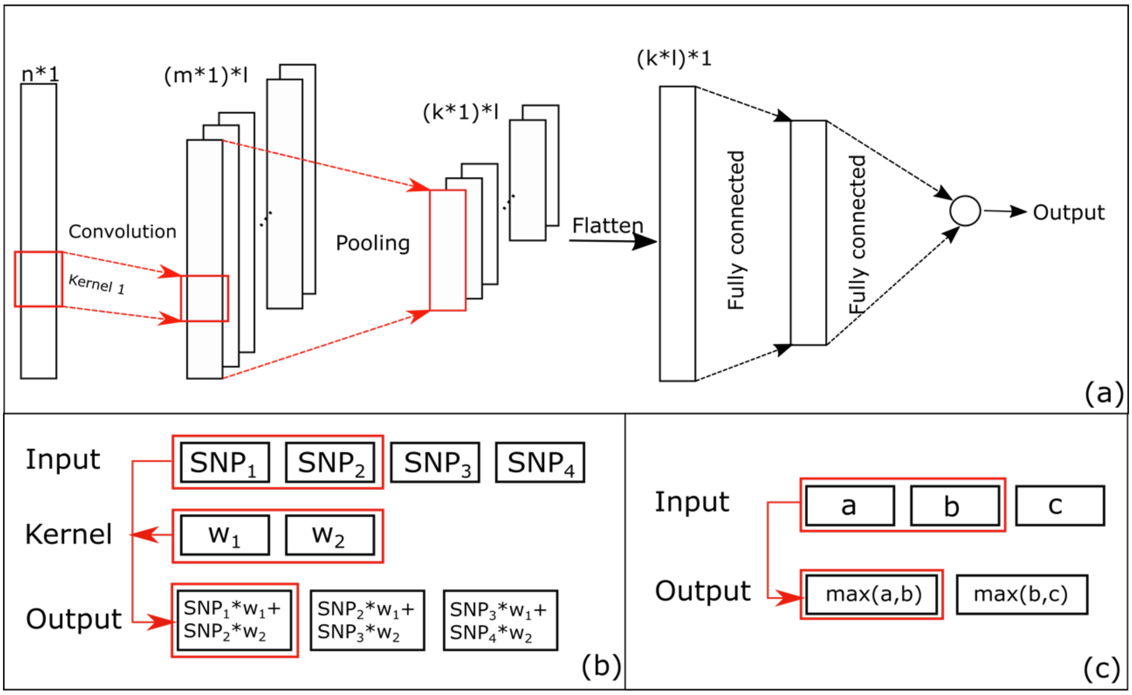

Supplement: Document S2. Article plus supplemental information [file mmc2.pdf]
